# Feasibility and acceptability of pooled sputum testing for tuberculosis: a multicountry qualitative study of the Start4All project

**DOI:** 10.1136/bmjopen-2026-120434

**Published:** 2026-07-30

**Authors:** Vibol Iem, Luan Nguyen Quang Vo, Andrew J. Codlin, Mosunmola Iwakun, Chimwemwe Kwanjo Banda, Douglas Akunda Okelloh, Cyrille Mbuli, Aline Santana Dosea, Victor Santana Santos, Kamal Ibne Amin Chowdhury, Tushar Garg, Jacob Creswell, Beate Ringwald, Ana I Cubas Atienzar, Rachel L Byrne, Tom Wingfield

**Affiliations:** 1 Centre for Tuberculosis Research, Liverpool School of Tropical Medicine, Liverpool, England, UK; 2 Friends for International TB Relief, Hanoi, Viet Nam; 3 Department of Global Public Health, Karolinska Institute, Stockholm, Stockholm County, Sweden; 4 Zankli Research Centre, Bingham University, Abuja, Nigeria; 5 Malawi-Liverpool-Wellcome Clinical Research Programme, Blantyre, Southern Region, Malawi; 6 Kenya Medical Research Institute, Centre for Global Health Research, Kisumu, Kenya; 7 Center for Health Promotion and Research, Bamenda, Cameroon; 8 Pharmacy Program, Department of Health, State University of Feira de Santana (UEFS), Feira de Santana, Bahia (BA), Brazil; 9 Department of Medicine, Federal University of Sergipe, Lagarto, Brazil; 10 Infectious Diseases Division, International Centre for Diarrhoeal Disease Research, Dhaka, Bangladesh; 11 Stop TB Partnership, Geneva, Switzerland; 12 Centre for Drugs and Diagnostics, Liverpool School of Tropical Medicine, Liverpool, England, UK; 13 Tropical and Infectious Diseases Unit, Liverpool University Hospitals NHS Foundation Trust, Liverpool, England, UK

**Keywords:** Tuberculosis, Implementation Science, INFECTIOUS DISEASES, Molecular diagnostics, Public health

## Abstract

**Abstract:**

**Objectives:**

Tuberculosis (TB) remains underdiagnosed in many high-burden countries due to high costs and limited availability of rapid molecular tests. Globally, only half of people with TB receive a molecular test as their initial diagnostic test. Pooled testing combines sputum samples from multiple individuals into a single molecular assay, offering cost savings by optimising existing resources, particularly in resource-constrained health systems. Although pooled sputum testing has now been recommended by the World Health Organization in 2026, evidence on real-world feasibility and acceptability for people with TB and healthcare providers remains limited. This study explored multisectoral stakeholder perspectives on the feasibility and acceptability of pooled sputum testing for TB.

**Design:**

Multicountry qualitative study using key informant interviews and focus group discussions. Data were analysed using a framework approach guided by established feasibility and acceptability models.

**Setting:**

Facility and community settings across seven high-TB-burden countries: Bangladesh, Brazil, Cameroon, Kenya, Malawi, Nigeria, and Viet Nam.

**Participants:**

Thirty-eight key informant interviews and 18 focus group discussions were conducted with National TB Programme (NTP) representatives, laboratory technicians, people with TB and international experts.

**Results:**

Pooled testing was considered feasible and acceptable in facility and community settings with greater confidence in countries with prior implementation experience. Feasibility depended on alignment with diagnostic algorithms and laboratory workflows, biosafety, training and clear standard operating procedures.

Acceptability operated at two levels: early NTP engagement with healthcare providers to secure professional buy-in and clear communication between healthcare providers and people undergoing testing to support understanding.

**Conclusions:**

Pooled testing was considered feasible and acceptable in settings with robust laboratory systems and routine practices. Readiness varied across countries, with some suitable for rapid implementation and scale-up while others required system strengthening. In settings perceived as having suitable infrastructure and readiness, pooled testing was viewed as a pragmatic strategy to optimise molecular TB testing.

**STRENGTHS AND LIMITATIONS OF THIS STUDY**

- This multicountry qualitative study included diverse multisectoral stakeholders across seven countries with high tuberculosis (TB), TB/HIV or drug-resistant TB burden, allowing comparison of pooled testing across different health system contexts.
- Data collection and analysis were guided by established conceptual frameworks of feasibility and acceptability, providing a structured and transparent analytical approach.
- Use of both key informant interviews and focus group discussions enabled triangulation of perspectives across policy, laboratory, clinical and people with lived experiences.
- People with TB were under-represented relative to healthcare providers and key informants, limiting the breadth of perspectives from people with lived experience.
- Variations in research capacity and prior experience with pooled testing of participants from across the seven countries may have led to conflicts between some participants’ theoretical perceptions of pooled testing versus others’ real-world reporting of implementation grounded in practice.

## Introduction

 Tuberculosis (TB) is the leading infectious disease killer globally, which caused an estimated 10.7 million people to fall ill and 1.23 million deaths in 2024.^1^ Despite major progress in TB prevention and care, millions of people with the disease remain undiagnosed or experience substantial delays in diagnosis and treatment, sustaining transmission and preventable mortality^2^ and underscoring the need for innovative approaches to expand access to rapid and accurate testing.^3 4^

Low-complexity nucleic acid amplification tests (LC-NAATs) are WHO‐recommended rapid diagnostic tests (WRD) for diagnosing TB and rifampicin resistance.^5^ Although LC-NAATs such as GeneXpert^6^ have significantly improved accuracy and drug resistance detection, their uptake remains limited. In 2024, only 54% of all people diagnosed with TB received a WRD as the initial diagnostic test, and only eight of the 30 high TB burden countries reported that more than 50% of their TB diagnostic sites had access to these tools.^1^ This reflects systemic challenges including cost, infrastructure gaps and insufficient laboratory capacity, particularly at the primary healthcare level.^7^

One approach to maximise the use of existing diagnostic capacity is pooled sputum testing, in which sputum samples from multiple individuals are combined and tested together using a single assay.^8 9^ If the pool tests negative, all individuals are considered negative; if the pool tests positive, individual tests on the same original sample are needed to identify those with disease. By reducing the total number of molecular tests required, this strategy can extend access to LC-NAATs, particularly in settings with high testing volumes and/or low TB prevalence.^10–12^ Studies evaluating pooled sputum testing using molecular diagnostics have reported sensitivities of 87.5%–98%, depending on pool size and assay used and specificities of 99%–100% compared with individual testing, alongside improved efficiency and substantial cost savings.^8 13^ Despite promising performance and efficiency gains,^14^ this strategy has not been widely adopted by National TB Programmes (NTP) and informed the WHO's 2026 recommendation on pooled sputum testing as part of updated TB diagnostic guidelines.^15^ However, evidence remains limited on how pooled sputum testing integrates into routine diagnostic workflows and on its acceptability among policy decision-makers, healthcare providers and people affected by TB.

This qualitative study, conducted as part of the Start4All project^3^ addressed these gaps by exploring key stakeholder perspectives on the feasibility and acceptability of pooled sputum testing across diverse epidemiological and programmatic settings, to inform policy decisions and practical implementation guidance.

## Methods

### Study design and analytical focus

The broader Start4All qualitative study^3^ explored the feasibility and acceptability of several TB tests and combinations of tests, including TB lipoarabinomannan assay, C-reactive protein tests (both rapid and machine-based), computer-aided detection for chest X-ray and molecular pooled sputum testing, to identify the most promising diagnostic algorithm for further evaluation. A successful algorithm was defined as one that demonstrates strong diagnostic performance, increases accurate identification of people with TB while excluding those without the disease, provides good value for money to the health system and is feasible to deliver and acceptable to those providing and receiving testing.

This manuscript represents a prespecified secondary analysis focusing on pooled sputum testing. Data segments relevant were extracted and reanalysed independently to generate evidence that informed the WHO's subsequent 2026 recommendation on pooled sputum testing for tuberculosis diagnostics.^15^ This manuscript was prepared in accordance with the Standards for Reporting Qualitative Research (SRQR).^16^ A completed SRQR checklist is shown in online supplemental table 1.

### Qualitative approach and conceptual framework research paradigm

Data were analysed using a thematic framework analysis. The analysis was guided by an integrated conceptual framework, which combined established feasibility and acceptability frameworks adapted to the scenario of pooled testing.

Feasibility was informed by frameworks from Klaic *et al,*^17^ Barker *et al*^18^ and French *et al*^19^ and defined as the extent to which pooled testing could be integrated into existing laboratory systems without compromising clinical standards or equity of access.

Acceptability was determined by Sekhon *et al*’s Theoretical Framework of Acceptability,^20^ which explores perceptions of people delivering or receiving an intervention on its appropriateness, based on cognitive and emotional responses.

**Figure 1:**
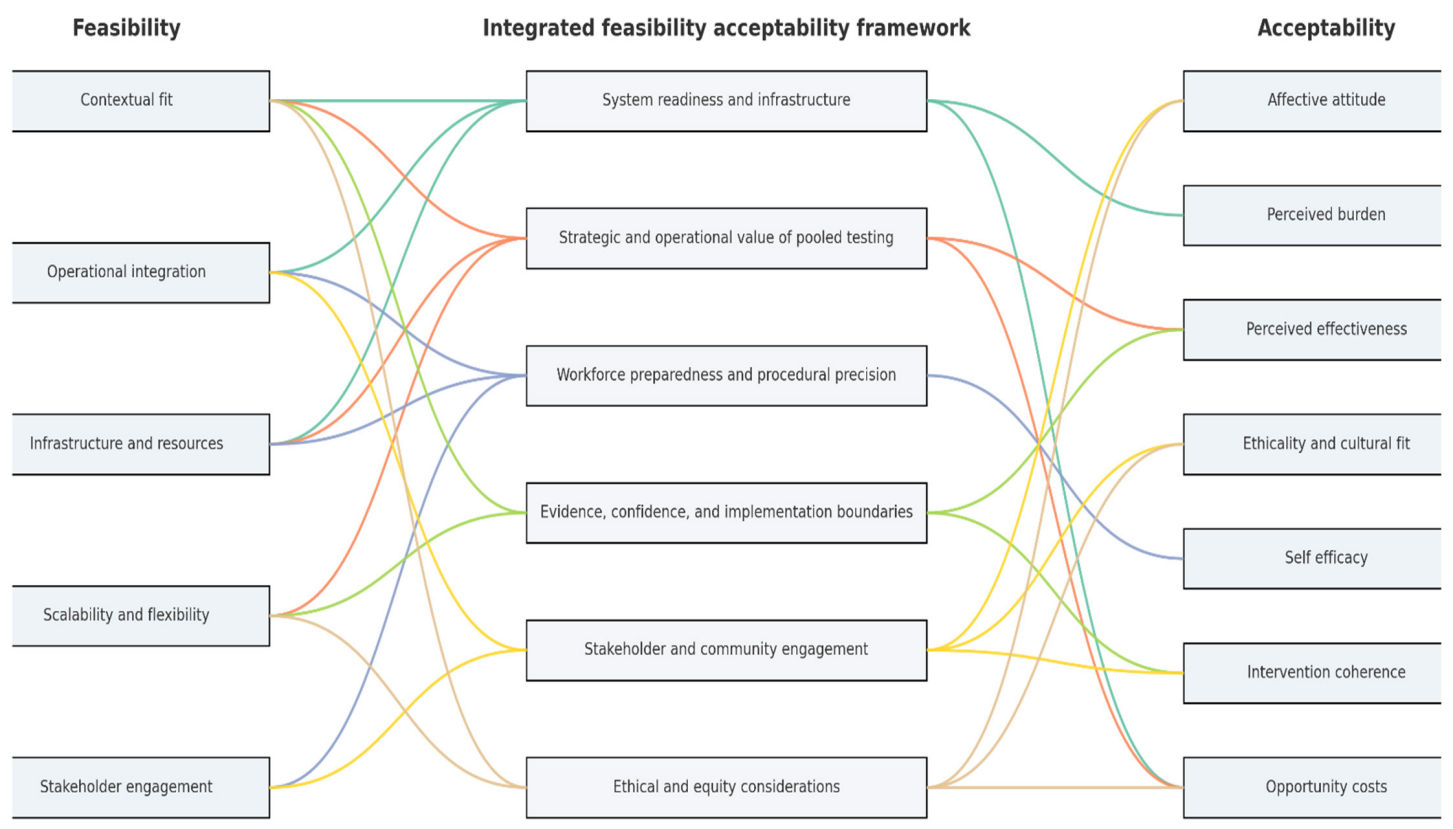
Integrated feasibility and acceptability framework for pooled testing.

The full conceptual framework, including all feasibility and acceptability domains and their operationalisation, is described in figure 1 and online supplemental methods.

### Researcher characteristics and reflexivity

The research team included individuals with expertise in TB diagnostics, implementation science and qualitative research. Several members, including the corresponding author and senior author, had prior experience with pooled testing and were generally supportive of its potential benefits, which provided contextual insight but also posed a risk of positive bias. To mitigate this, data collection tools explicitly probed both perceived advantages and concerns, interviewers actively sought dissenting views and coding was undertaken by analysts with varying levels of involvement in pooled testing. Regular multidisciplinary discussions throughout data analysis encouraged researchers to critically reflect on how their prior experience and assumptions might influence interpretation, and to assess whether emerging interpretations were supported by the data, thereby enhancing the trustworthiness of the qualitative findings. These measures aimed to ensure that analysis was grounded in participants’ perspectives rather than researchers’ prior expectations.

### Context

This qualitative study was conducted as part of the Unitaid-funded Start4All initiative^3 4^ across seven countries identified by the WHO as having a high burden of TB, and/or multidrug-resistant TB and/or TB–HIV coinfection: Bangladesh, Brazil, Cameroon, Kenya, Malawi, Nigeria and Viet Nam. These countries were purposively selected to reflect diverse epidemiological profiles, economic contexts, diagnostic capacity, TB programme maturity and health system structures, including variation in decentralisation and workforce capacity.

Across sites, familiarity with pooled testing varied. At the time of data collection, Cameroon had integrated pooled testing into routine TB diagnostics in some sites, while Brazil, Nigeria and Viet Nam were piloting or evaluating the approach. In Bangladesh, Kenya and Malawi, pooled testing was still at the exploratory or planning stage. These differing stages of adoption provided valuable contextual variation across settings, shaping stakeholder perspectives on the feasibility, acceptability and potential for scale-up of pooled testing.

### Sampling strategy

A purposive sampling strategy was used to capture diverse perspectives on the feasibility and acceptability of pooled testing across roles and settings. Eligibility was restricted to adults ≥18 years who were able to provide informed consent.

For international key informant interviews (KIIs), participants were purposively selected to capture global perspectives on TB diagnostics, market considerations and implementation at scale.

National level key informants were selected to represent clinical, laboratory and programme perspectives of stakeholders involved in TB diagnostic service delivery.

Focus group discussions (FGDs) with healthcare providers were designed to explore operational feasibility, workflow integration, perceived challenges and enablers of pooled testing in routine practice.

FGDs with people with TB were designed to capture lived experiences of diagnostic processes, perceived burden, understanding of pooled testing and acceptability of testing procedures. Sampling continued until thematic saturation was reached, assessed through ongoing review of KII and FGD data to determine when no new themes relevant to feasibility and acceptability emerged.

Further details on sampling procedures and participant selection are provided in the online supplemental methods.

### Ethical issues

Ethical approval for the study has been provided by LSTM Research Ethics Committee, WHO Ethics Review Committee and by each participating country’s institutional review board (online supplemental table 2).

Further details on consent procedures and participant protections are provided in the online supplemental methods.

### Data collection methods

Research was conducted within Start4All study sites, NTP offices and public sector health facilities. Data collection took place concurrently across the seven countries without a fixed sequence of sites or activities.

KIIs and FGDs data were collected between 1 October 2024 and 30 June 2025. KIIs were conducted individually, in person or virtually, while FGDs were held in person with 6–8 participants in familiar, convenient settings such as TB clinics or community centres across the seven countries.

### Data collection instruments and technologies

A semistructured topic guide with open-ended questions was used in all interviews and discussions to ensure consistency while allowing flexibility for emerging themes. Guides were developed with country partners based on predefined feasibility and acceptability frameworks, piloted with international key informants and refined through debriefing sessions and feedback. They were prepared in English and translated into local languages as needed for contextual clarity and cultural appropriateness.

### Units of study

Six international KIIs were conducted virtually using Microsoft Teams. Thirty-two national-level KIIs were conducted in person across the seven countries.

A total of 18 FGDs were held across all sites, including 70 healthcare providers and 48 people with TB.

Detailed breakdowns by country and participant characteristics are provided in online supplemental tables 3–5, with further details in the online supplemental methods.

### Data processing and analysis

All audio recordings were transcribed verbatim in the original language by trained research staff. In Bangladesh, Brazil, Cameroon and Viet Nam, transcripts were professionally translated into English, preserving the original meaning. Each transcript was reviewed for accuracy and anonymised, removing any identifying information to ensure participant confidentiality.

All transcripts were imported into NVivo V.14 for coding.^21^ Country research teams initially coded transcripts using a predefined codebook aligned with the feasibility and acceptability frameworks. The first author (VI) then reviewed and recoded transcripts where necessary to ensure consistency across countries. Regular consensus meetings with the country qualitative research focal points were held throughout the analysis to discuss coding decisions, resolve discrepancies and iteratively refine the codebook, thereby promoting consistent application of the coding framework across countries. The final codebook is presented in table 1.

**Table 1:** Codebook

Theme	Code	Definition
System readiness and infrastructure	1.1 Enabling laboratory infrastructure	Existing systems and stable infrastructure support smooth integration of pooled testing.
	1.2 Organisational and workflow burden	Additional pooling steps increase coordination demands and risk of error.
	1.3 Linkage to care as part of readiness	Delays in communicating results or initiating treatment undermine pooled testing impact.
Strategic and operational value of pooled testing	2.1 Strategic expansion of diagnostic coverage	Pooled testing viewed as a cost-effective strategy to extend molecular testing reach and strengthen diagnostic networks.
	2.2 Efficiency in low-positivity settings	Pooled testing considered most suitable where positivity rates are low, allowing cartridge savings without disrupting routine workflow.
Workforce preparedness and procedural precision	3.1 Training and supervision for quality assurance	Clear SOPs, defined roles and regular supervision ensure procedures consistency and make pooled testing feel routine.
	3.2 Prior experience strengthens coordination	Previous exposure to pooled testing approaches improves understanding and team coordination.
	3.3 Procedural precision and workload sensitivity	Pooled testing relies on meticulous labelling and traceability; heavy workload or fatigue can compromise accuracy.
Evidence, confidence and implementation boundaries	4.1 Programme validation builds confidence	Prior evaluation, pilot implementation or published evidence increases trust in pooled testing.
	4.2 Dilution sensitivity concerns in specific populations	Risk of false negatives in children, people living with HIV or previously treated individuals; pooled testing should be used selectively.
	4.3 Regulatory and manufacturer deviation concern	Hesitation to deviate from manufacturer instructions due to fears of reduced validity or non-compliance.
Stakeholder and community engagement	5.1 Communication intensity and change management	Pooled testing requires proactive, repeated explanation due to departure from the one-person-one-test model.
	5.2 Trust in providers explanation	Acceptability depends on confidence in healthcare providers’ clear and reassuring communication.
	5.3 Perceived benefit and timeliness	Acceptability increases when pooled testing is seen to reduce delays and support earlier treatment.
Ethical and equity considerations	6.1 Pragmatic equity argument	Pooled testing viewed as a fair and practical way to extend access where universal molecular testing is unaffordable.
	6.2 Tension between efficiency and universal detection	Concerns that focusing on cost savings may divert attention from diagnosing and treating all people with TB.

SOPs, standard operating procedures; TB, tuberculosis.

Further details on transcription, translation, coding procedures and analytic synthesis are provided in the Supplementary Methods.

### Patient and public involvement

People with TB were involved in the conduct of the study through participation in FGD and interviews that informed the assessment of feasibility and acceptability. They were not involved in the design or dissemination of the research.

## Results

The key feasibility and acceptability themes are summarised in table 1.

### System readiness and infrastructure

Across settings, respondents emphasised that the feasibility of pooled testing depended on existing infrastructure and organisational capacity. In facilities with established GeneXpert systems, stable power supply, adequate biosafety, sufficient workspace and reliable sample tracking, pooled testing could be integrated into routine workflows with minimal disruption.

GeneXpert pooled testing can be feasible even at the primary healthcare level. It doesn’t necessarily require specialised expertise. (International KII2, Female, Market shaping specialist)

However, others highlighted that pooled testing could place additional demands on laboratory organisation and workflow management. These included extra steps for sample handling, tracking and potential retesting, which could strain existing systems and increase the risk of errors in less well-resourced or high-throughput settings.

… adding new tasks will make organisation much more complicated, especially when working in the community and coordinating schedules. Manpower and finances are the major issues … and will certainly be barriers. (Viet Nam FGD with healthcare providers)

Some healthcare providers noted that even well-equipped laboratories have limited value if testing is not effectively linked to treatment. Delays in communicating results or initiating care could undermine pooled testing, meaning system readiness required not only equipment and trained staff but rapid translation of results into clinical action.

We are doing so many tests, but if the linkage is not strong, if the treatment is delayed, then what is the point? (Bangladesh FGD with healthcare providers)

### Strategic and operational value of pooled testing

NTP representatives and policymakers viewed pooled testing as a strategic, cost-effective way to expand diagnostic coverage. They saw it as part of efforts to strengthen molecular testing networks, extend services to lower-tier facilities and improve testing reach and sustainability within existing health systems, supporting efficiency and equity goals.

In primary healthcare settings, pooled testing is a method that should be encouraged in low-income settings like ours, where we are always having stock-outs. It’s economical and helps to screen more people with the same resources. (Cameroon KII1, Female, Programme/Technical Director)

Laboratory managers and staff emphasised that pooled testing works best in facilities where TB positivity rates are generally low, allowing laboratories to maintain both accuracy and efficiency, although no specific positivity threshold was identified. From their perspective, the key feasibility consideration was practical: ensuring that routine testing could continue smoothly without overloading machines or wasting cartridges.

If most [pooled samples] turn out negative, it is good for us … we save a lot of cartridges … it is something that is worth trying. (Kenya KII2, Male, Laboratory staff)

### Workforce preparedness

Healthcare providers and laboratory technicians reported increased proficiency in pooled testing once clear procedures, defined roles and regular supervision were in place. Familiarity with testing workflows, clear procedures and concise guidance would help make pooled testing feel routine rather than experimental.

After training, everyone knew what to do. We just followed the SOP, and it went smoothly. (Kenya FGD with healthcare providers)

Individuals with prior experience, particularly from SARS-CoV-2 pooled testing during the COVID-19 pandemic, reported greater team confidence and coordination. Familiarity with similar approaches helped staff understand the logic of pooled testing and reduced hesitation in handling multiple samples.

This is something that was used during the COVID time, where they pooled samples to minimise testing reagents. (Malawi KII3, Male, TB technical advisor)

Pooled testing was described as relying less on advanced expertise and more on meticulous organisation. Participants stressed that careful labelling and traceability were essential but warned that heavy workloads and fatigue could compromise these safeguards.

You have to have a very attentive professional who pays attention to the details. You have to collect several samples, then you can separate which samples are which, which were in that pool, so you can repeat the test. (Brazil KII4, Female, TB expert consultant)

### Evidence, confidence and implementation boundaries

Acceptability and feasibility of pooled testing was greater where it had been previously evaluated or implemented. Programme validation, pilot studies and published evidence from other settings reinforced perceptions that pooled testing was reliable and aligned with existing diagnostic strategies.

Pooled testing has already been validated by the programme and shown to be effective. (Cameroon KII2, Male, NTP coordinator)

Concerns were raised about reduced sensitivity in individuals with low bacterial loads, including children, people living with HIV and those with prior TB treatment. Participants warned that weak positive individual test being false negative pooled test could be missed and advised using pooled testing selectively in low positivity settings while retaining individual testing where sensitivity is critical.

I’ve encountered a situation where a sample run individually showed very low TB [but] when it was pooled, it came out negative. So, I think they shouldn’t just rely on pooled sampling. (Nigeria FGD with healthcare providers)

Other laboratory technicians expressed concerns not only about dilution effects but also about deviating from manufacturer recommended protocols. For them, pooled testing raised issues of regulatory compliance and test validity, reflecting hesitation to adapt molecular assays beyond approved instructions even if programmatically supported.

We may have false or discordant results because the methodology is not followed according to the manufacturer’s recommendation. (Brazil KII2, Female, TB laboratory specialist)

### Stakeholder and community engagement

Pooled testing was seen as requiring deliberate communication, as it departs from the familiar one-person-one-test model. Participants emphasised the need for clear messaging and proactive change management, particularly for older adults and those less familiar with new technologies.

The real challenge is getting the elderly involved, as they may not fully understand the process with new technologies. […] Effective coordination should involve extensive communication beforehand, with more than one meeting to ensure successful implementation. (Viet Nam FGD with healthcare providers)

Among people with TB, acceptability was described as dependent less on technical details and more on trust in healthcare providers’ explanations and reassurance.

If [healthcare providers] explain it very clearly, people would support it. (Brazil FGD with people living with TB)

Acceptability was also linked to perceived effectiveness. Stakeholders noted that people seeking care often prioritise speed and tangible outcomes, and that pooled testing could be viewed favourably when it was understood to reduce delays and facilitate earlier treatment.

Patients want quick, ready solutions. […] The public will appreciate a system where they can get tested and treated efficiently without unnecessary delays. (Bangladesh KII2, Male, Programme Coordinator)

### Ethical and equity considerations

Several respondents reflected on the broader ethical and equity implications of pooled testing. They explained that while universal testing with GeneXpert remains ideal, it is often financially unsustainable, and pooled testing offers a pragmatic compromise that can extend access without compromising fairness.

It’s just not feasible and we can’t afford to test everyone with GeneXpert. Pooled testing is a really good alternative, and we hope to gather good evidence to show other countries. (International KII2, Female, Market shaping specialist)

Some healthcare providers questioned prioritising cost efficiency while the TB burden remains high and many people are undiagnosed. They argued that introducing pooled testing now risks diverting attention from detecting and treating everyone, suggesting such optimisation may be more appropriate as countries approach elimination.

We should not be talking of cost at this stage … when we have drastically brought down TB in the population, then we can talk about pooled testing. But for now, let’s actively get these people and treat them. (Nigeria FGD with healthcare providers)

## Discussion

This qualitative study explored views on pooled sputum testing among policy makers, healthcare providers and people seeking care across seven high-burden countries, and found marked variation across settings, shaped by differences in laboratory capacity, service organisation and overall health system readiness.

Feasibility and acceptability appeared to follow a maturity gradient across countries. In Cameroon, where pooled testing had been integrated into routine NTP practice,^22^ it was described as efficient and uncontroversial. In contrast, in countries at conceptual or pilot stages, enthusiasm was often accompanied by uncertainty about SOPs, follow-up procedures and operational safeguards. This progression from uncertainty in pilot settings to routine practice reflects processes described in the Normalisation Process Theory,^23^ whereby interventions become more acceptable as collective understanding, operational workability and confidence strengthen over time.

Where electricity supply, biosafety standards, sample tracking and linkage to care were already strong, pooled testing was considered easy to implement with little disruption. Where these foundations were fragile, the additional organisational steps exposed weaknesses. This suggests that pooled testing relies on strong systems and may reveal weaknesses where they exist. These findings reinforce broader lessons from molecular diagnostic rollouts, where test performance alone did not guarantee programme impact without adequate integration into national algorithms, consistent with WHO guidance for introducing new diagnostics.^24^

Stakeholders viewed pooled testing as strategically valuable only under specific epidemiological and operational conditions. Its efficiency gains were perceived to depend on low positivity rates and manageable testing volumes, aligning with modelling evidence showing diminishing returns above approximately 30% positivity.^13^ This indicates that pooled testing is not inherently superior to individual testing and its value is context dependent. Policy guidance should therefore emphasise selective deployment,^24^ supported by decision-support tools that help programmes determine when pooled testing is appropriate and when individual testing should remain standard practice.

Another important insight is that pooled testing relies more on procedural discipline than advanced technical expertise. Participants linked feasibility and acceptability to clarity of roles, clear SOPs and supervision rather than specialised skills, a nuance that is rarely captured in quantitative performance studies. At the same time, heavy workloads, staff turnover and informal workarounds posed risks to procedural consistency. Similar vulnerabilities have been observed when rapid diagnostic scale-up outpaces training capacity.^25^ Quality assurance mechanisms for pooled testing must therefore prioritise traceability and documentation, and be integrated into national monitoring frameworks.^26^

Evidence and trust emerged as central to acceptability. Participants frequently cited studies from Brazil,^10^ Cameroon^22^ and Nigeria^12^ showing high agreement between pooled and individual results, which strengthened confidence that sensitivity was not compromised by sample dilution. However, trust in evidence did not eliminate normative caution. In practice, concerns about reduced sensitivity can be addressed through selective implementation strategies. For example, the Cameroon NTP pooled testing strategy explicitly excludes people living with HIV and people previously treated for TB from pooled testing, with these individuals instead undergoing individual molecular testing as their initial TB test. Challenges persisted for populations with low bacterial loads, and some laboratory technicians expressed concerns about deviating from manufacturer-recommended protocols. This highlights that implementation boundaries are shaped not only by empirical data but by professional norms and perceived responsibility for diagnostic certainty.^27^ Clear regulatory positioning and guidance on appropriate subpopulations will therefore be essential to avoid inconsistent application across sites.

Unlike other diagnostic interventions, pooled testing alters the one-person-one-test logic that people seeking care and healthcare providers expect. Misunderstandings about positive pools and retesting largely reflected communication gaps rather than methodological flaws. Other studies showed that misunderstanding of procedures can undermine people's trust in healthcare providers and the diagnostic process.^27^ Acceptability among people with TB depended less on technical details and more on trust in healthcare providers and perceived effectiveness. Clear, person-centred explanation of result interpretation is therefore crucial for pooled testing implementation success.^28^ Change management and community engagement should be embedded within implementation guidance rather than treated as peripheral activities.^24^

Stakeholder engagement was another determinant for successful implementation of pooled testing. Healthcare providers expressed concerns that an intervention perceived as externally imposed, without adequate local consultation, could undermine local ownership and hinder uptake. Where NTPs had endorsed pooled testing and involved implementers and communities early, acceptability and compliance were much stronger. These experiences underline the importance of participatory planning and change management.^22^ Future implementation guidance should provide templates for stakeholder mapping, communication materials for community engagement and structured feedback mechanisms to maintain collaboration between national and subnational levels.

### Study limitations

While the study captured rich perspectives, it also had some limitations.

First, our data exemplified all feasibility and acceptability domains within the robust conceptual framework that guided our analysis. However, people with TB were under-represented in the sample compared with key informants and healthcare providers.

Second, differences in research capacity and experience across the seven countries may have introduced variation in depth and quality of data. While varied experience enriched insights into how acceptability evolves with scale and familiarity, views from those without direct experience were sometimes theoretical rather than practice based.

Third, collaborative analysis and shared codebook development supported consistency, but the professional involvement of investigators in TB diagnostics may have biased interpretation.

## Conclusions

Pooled testing for TB was considered feasible and acceptable, offering a practical way to optimise molecular testing capacity and equity of access. Feasibility increased with system readiness and clear guidance, while acceptability relied on trust, communication and programme ownership. Key priorities for scale-up include clear planning on where, for whom and why pooled testing is implemented, alongside robust monitoring and evaluation to assess performance and refine delivery during implementation. These insights can inform the development of an implementation manual and toolkit, supporting a safe, efficient and person-centred roll-out.

## Supplementary material

10.1136/bmjopen-2026-120434 online supplemental file 1

10.1136/bmjopen-2026-120434 online supplemental file 2

## Data Availability

Data are available upon reasonable request. All data relevant to the study are included in the article or uploaded as supplementary information.
