# Supplementary material for: Feasibility and acceptability of pooled sputum testing for tuberculosis: a multicountry qualitative study of the Start4All project
Source: BMJ Open. 2026 Jul 30;16(7):e120434. doi: 10.1136/bmjopen-2026-120434 (PMC13436116; doi:10.1136/bmjopen-2026-120434)
Supplement: Supplementary file 1 [file bmjopen-16-7-s001.pdf]

Supplementary Methods

Qualitative approach and conceptual framework research paradigm

A qualitative descriptive approach was selected to enable a structured yet flexible exploration of stakeholders' views on pooled sputum testing for TB across diverse countries, settings, roles, and experiences. This design suited the study's aim to inform policy and guideline development by identifying participants' perspectives on context-specific barriers, enablers, and practical insights of pooled sputum testing for TB. Guiding frameworks, selected iteratively by consensus amongst the study team, anchored the analysis in implementation science while permitting the identification of themes not predefined within the frameworks.

The conceptual framework for this study adapted tested feasibility and acceptability frameworks developing an integrated framework tailored to pooled testing. Feasibility drew on definitions by Klaic *et al.,*[16] Barker *et al.,*[17] and French et al.[18] addressing key factors for implementation and scale-up. These frameworks emphasise health system integration, service complexity, and behavioural influences, offering complementary perspectives applicable to diverse TB settings. Feasibility was defined as the extent to which pooled testing could be integrated into existing laboratory systems without compromising clinical standards or equity[16-18]. Five domains were considered: contextual fit (alignment with policies and needs), operational integration (compatibility with care pathways), infrastructure and resources (diagnostics, workforce, logistics), scalability and flexibility (adaptability and expansion potential), and stakeholder engagement (local involvement and national alignment).

Acceptability was determined by Sekhon *et al.*'s Theoretical Framework of Acceptability,[19] which explores perceptions of people delivering or receiving an intervention on its appropriateness, based on cognitive and emotional responses. Although originally designed for healthcare interventions, this framework has been widely applied in implementation studies of novel diagnostic or screening approaches[27,28] and is therefore deemed appropriate for assessing stakeholders' perceptions of pooled testing workflows. It includes seven domains: affective attitude (emotional response), perceived burden (effort or inconvenience), perceived effectiveness (belief in purpose), ethicality and cultural fit (value alignment), self-efficacy (confidence in ability), intervention coherence (understanding of how and why it works), and opportunity costs (trade-offs in time, resources, and value).

Elements from these frameworks were combined into an integrated framework for feasibility and acceptability of pooled testing to clarify how system readiness, operational value, workforce preparedness, evidence boundaries, stakeholder engagement, and ethical considerations intersect in pooled testing implementation. Supplementary Figure 1 presents this integrated framework, illustrating how domains from the feasibility and

acceptability frameworks informed a shared set of analytical themes used throughout data collection and interpretation.

Sampling strategy

We used a purposive sampling strategy to capture diverse perspectives on the feasibility and acceptability of pooled TB testing across roles, settings, and countries. Sampling aimed to include participants with direct experience of TB diagnostic pathways and those involved in programme oversight, implementation, and policy decision making.

Eligible participants were aged 18 years or older; individuals who declined or were unable to provide informed consent were excluded. Participants were purposively selected to ensure diversity in gender, age, professional role, and experience.

For international KIIs, participants were purposively selected from funder, manufacturer, and policy networks to capture global perspectives on TB diagnostics, market considerations, and implementation at scale, and were approached via email by the central study team.

For national level KIIs, participants were purposively selected to represent clinical, laboratory, and programme perspectives, including Ministry of Health officials, National TB Programme representatives with programme management or policy responsibilities, and frontline healthcare providers involved in TB diagnostic service delivery.

For FGDs with healthcare providers, participants were sampled from staff involved in TB screening, sample collection, laboratory processing, and clinical management during Start4All implementation. These discussions were designed to explore operational feasibility, workflow integration, and perceived challenges and enablers of pooled testing in routine practice.

For FGDs with people with TB, participants were purposively sampled from individuals receiving TB diagnostic or treatment services at Start4All participating health facilities. These discussions aimed to capture lived experiences of diagnostic processes, perceived burden, understanding of pooled testing, and acceptability of testing procedures.

FGDs were used to capture shared experiences, collective norms, and points of agreement or tension among participants involved in TB diagnostic processes, allowing interaction between participants to surface operational nuances that may not emerge in individual interviews. KIIs were used to obtain in-depth, individual perspectives from participants with specific technical, managerial, or policy expertise, and to enable candid discussion of sensitive or system-level issues that may not be appropriate for group settings. The combined use of FGDs and KIIs was intended to generate complementary insights across different roles and levels of the TB diagnostic system.

Sampling continued until a priori thematic saturation was reached, assessed through interim analysis of interview and discussion data. The number of KIIs and FGDs conducted

in each country therefore varied according to stakeholder availability and the point at which feasibility and acceptability domains were sufficiently exemplified.

Ethical issues

Ethical approval for the study has been provided by LSTM Research Ethics Committee (REC), WHO Ethics Review Committee (ERC), and also by each participating country's institutional review board (Supplementary Table 2).

All participants were provided with detailed information about the study purpose, procedures, and their rights. Written informed consent was obtained from all participants prior to data collection, in accordance with the study and each country's ethical approval requirements. Participation was entirely voluntary, and individuals could withdraw at any time without consequence.

Recruitment was coordinated by country teams via phone or email in line with local procedures. Local research teams ensured that recruitment, consent, and data collection procedures were culturally appropriate and aligned with local norms.

Units of study

Six international KIIs were conducted virtually using Microsoft Teams. Thirty-two national-level KIIs were conducted in-person at key informant offices: four interviews each in Bangladesh, Brazil, Kenya, and Malawi; five in Nigeria and Viet Nam; and six in Cameroon.

A total of 18 FGDs were held in person with 6–8 participants in familiar, convenient settings such as TB clinics or community centres across the seven countries. In six countries, one FGD was held with healthcare providers and one with people with TB. In Viet Nam, one FGD was held with people with TB and five FGDs were conducted with healthcare providers. Across all sites, FGD participants included 70 healthcare providers and 48 people living with TB.

Variation in the number of KIIs and FGDs across countries reflected our sampling approach, which involved continuing recruitment until identified acceptability and feasibility domains were exemplified[29] in each setting. Country teams therefore adapted the number of interviews and discussions according to stakeholder availability and when level of saturation[29,30], which explains differences such as more FGDs in Viet Nam. Detailed demographic characteristics are provided in Supplementary Tables 3, 4 and 5.

Data processing and analysis

All audio recordings from KIIs and FGDs were transcribed verbatim in the original language by trained research staff. Where needed, transcripts were professionally translated into English, preserving the original meaning. Each transcript was reviewed for accuracy and anonymised, removing any identifying information to ensure participant confidentiality.

All transcripts were imported into NVivo 14 for coding[20]. A predefined codebook reflecting the applied frameworks was employed across all transcripts, with additional codes introduced when data did not fit existing framework domains. This primarily deductive approach allowed systematic comparison across countries and stakeholder groups while facilitating new context-specific insights when they arose. Coding consistency was ensured through regular analyst meetings and iterative review of coded data supported by cross-site research team meetings to ensure alignment in code application and thematic interpretation. Analytical summaries were generated in each country and then synthesised to identify convergent and divergent perspectives. The final codebook is presented in Table 1.

Techniques to enhance trustworthiness

Trustworthiness was established following Korstjens et al.,[31] and Lincoln et al.[32] qualitative research criteria and related practical guidance. Measures were applied across the key dimensions of credibility, transferability, dependability, and confirmability.

Credibility was supported through triangulation of data sources and stakeholder perspectives across the seven countries, as well as peer debriefing between central and country teams to validate interpretations and resolve differing views.

Transferability was enhanced by providing detailed descriptions of each country's implementation context, participant groups, and health system structures to allow assessment of applicability to other settings.

Dependability was maintained through an audit trail documenting codebook development, coding decisions, and analytic summaries, enabling transparent tracing of how findings were derived.

Confirmability was strengthened by team-based analysis whereby multiple analysts across country and central teams independently reviewed transcripts, compared coding, and jointly resolved discrepancies. This collaborative approach ensured that interpretations were grounded in participant accounts, cross-validated across analysts, and not driven by any single researcher's assumptions.

Topic Guide for in-person and virtual Key Informant Interviews (~ 60-90 minutes total)

I. Introduction - Ground rules

Always remember that the participants are the experts, and they have information that you do not have but would like to learn.
Welcome and thank the interviewee for taking part in the interview and remind them that they have been asked to participate as their point of view is important.

- *Hello. Thank you for agreeing to do this interview. My name is [lead interviewer's name], and I'll be talking with you today. We are also pleased to be joined today by [research assistant/note taker].*
- *The interview will last about 1 hour.*

A. Consent form and need for consent and signature

- *Have you received the participant information sheet and had a chance to read it? Do you have any questions?*
- *Feel free to ask questions anytime during our conversation.*
- *If there's anything you prefer not to answer, just let me know, and we'll move on*
- *You can stop the interview at any time without needing to explain.*
- *Please remember that we want to know what you think and feel and that there are no right or wrong answers.*

B. Reinforce that the discussion will be anonymous

- *Everything you share will be kept confidential. Your name and organization will not be linked to anything you say and will remain anonymous at all times.*
- *Your responses will be written anonymously and reported in aggregate. No one will know how you, as an individual, responded in the final report.*
- *We'd like to record this session to help with summarizing and transcription. Recordings will be securely stored and destroyed after transcription, and no identifying information will be included in the notes.*
- *Is it OK if I record this interview today?* **[Turn on recording equipment]**

II. Background

Explain purpose of the interview, that it is meant to assess thoughts, impressions, information and feelings about Start4All tests and test combinations.

Start4All is a project that is funded by Unitaid and implemented by LSTM. The objective is to assess the performance of WHO-approved TB tests and test combinations in primary healthcare settings and among key and vulnerable populations, including those living in communities with high HIV prevalence, rural or informal settlements with poor access to health facilities, marginalized groups like refugees and nomads, and children.

By combining TB tests, we mean using tests sequentially to enhance accuracy and cost-effectiveness. The idea is to first use a screening test to identify likely TB cases, then follow up with a confirmatory test. A successful combination should be feasible to deliver, acceptable to users and providers, increase accurate TB diagnoses, exclude non-TB cases, and be cost-effective for the health system.

Use the visual/illustrative handout/summary that was given at the time of the recruitment and re-explain briefly what we mean by combinations of tests, giving some concrete examples graphically.

Today's interview focuses on TB test combinations for screening and diagnosis. We want your insights on the main barriers and enablers affecting their acceptability, feasibility, and scalability. This will help us ensure that TB services meet the needs of populations lacking access.

I'd like to begin by asking you some questions about your current job.

1. *What is your position and role at [organization]?*
2. *Can you tell me a bit about your work and experience as it relates to TB diagnosis and TB tests? (Probe particularly for aspects of current job that relate to Start4All)*
3. *What is your organization's experience with implementing new diagnostic strategies?*

III. Discussion/Questions

Thank you. Now I would like to discuss a little more in details acceptability of combination of tests, especially factors to consider for implementation.

a) Affective attitude - Feelings towards the intervention

1. *What do you think of the idea of combining different TB tests to perform a screening or triage step first and then, for those screening positive, a confirmatory test?*
   - ➔ *Possible probes if needed to support interviewee responses: What could be the benefits/drawbacks?*

b) Capability/Practicality

2. *How easy or complicated would it be to explain the concept of combination of TB tests to people attending primary healthcare facilities and how can these challenges be addressed?*

c) Burden

3. *How do you think different* ***health care workers*** *will perceive the use of combined TB testing methods? For example, healthcare workers can include nurses, radiographers, radiologists, laboratory technicians, physicians/doctors, and community health workers.*
   - ➔ *Possible probes using examples listed below if needed to support interviewee responses:*

     - How might nurses involved in TB screening view the implementation of these methods?
     - What could be the perspectives of radiographers who conduct chest X-rays (CXRs) in this context?
     - What challenges or benefits might radiologists see with this approach?
     - How would laboratory technicians working in district-level labs perceive the use of combined TB tests?
     - How might physicians/doctors interpret the use of combined tests in their clinical practice?
     - What are the thoughts of community health workers on implementing these methods?
     - Foreseen challenges, high workload, limited number of staff, technical capability

4. *How do you think* ***people with presumptive TB*** *will perceive/accept the use of combinations of TB tests?*

- ➔ *Possible probes if needed to support interviewee responses: stigma, discrimination, time (including waiting time), costs, trust in the test, having to give more than one sample, types of sample*

d) Social influences

5. *What social or cultural factors might influence the acceptance and use of the TB test combinations among healthcare workers? Among health seeking people?*

6. *How can these social or cultural factors be leveraged or mitigated to ensure successful implementation?*

e) Ethicality

7. *All the TB tests are WHO-approved for safety and effectiveness for certain groups of population. Do you have any ethical concerns about using a combination of these tests, such as patient consent, privacy, or potential burden on patients?*

f) Intervention coherence / Contextual Fit

Now I would like to talk about the contextual fit and how appropriate/relevant do you think combinations of TB tests is for primary healthcare settings or for outreach ACF campaigns (in remote hard to reach areas, or overcrowded slums for example).

8. *What are your thoughts on the use of blood tests (e.g., CRP) in these settings? How do finger prick and venipuncture methods compare for use in adults and children? What factors (e.g., lab needs, materials, skillsets) influence the choice of method in these settings?*

9. *What do you think of the practicality and feasibility of collecting urine and the use of urine tests (including LAM) for adults and children in those settings?*
   Use these prompts as optional tools to aid understanding or provide examples but adapt or skip them based on the interviewee and interview context.

   - ➔ *Possible probes: Urine Collection Process:*
     - Toilet facilities are available to patients? Are they clean and private?
     - Issues with dehydration affecting sample collection?
     - Are there concerns about urine leakage due to improper sealing?

   - ➔ *Possible probes: Challenges in Urine Collection:*
     - Do patients struggle with directing urine into containers, especially with limited water or toilet paper?

- How does timing and the time required to provide a sample affect the process?
- What aids could improve the collection process?
- What other challenges have been encountered with other urine-based tests like pregnancy or Urinary Tract Infection (UTI) testing?

10. *What do you think of the idea of collecting stools samples for adults and children in those settings?*

Use these prompts as optional tools to aid understanding or provide examples but adapt or skip them based on the interviewee and interview context.

- ➔ *Possible probes - Sample Production:*
  - Can individuals produce stool samples on demand, or would multiple clinic visits be needed?
  - Handling and Storage: How feasible is it for patients, especially parents, to handle and store stool samples properly before bringing them to the clinic?
  - Comfort and Privacy: How can clinics ensure comfort and privacy during the collection process?
  - Parental Involvement: For children, would stool collection require multiple visits or significant parental involvement? Are guardians comfortable managing and transporting stool samples?

11. *What do you think of the idea of collecting gastric aspirates samples in those settings, especially for children?*

Use these prompts as optional tools to aid understanding or provide examples but adapt or skip them based on the interviewee and interview context.

- ➔ *Possible probes*
  - In your experience, is it typically necessary for patients to be admitted to the hospital for gastric aspirate sampling, or can this procedure be conducted in an outpatient setting?
  - How common is it for healthcare professionals to be trained in placing nasogastric (NG) tubes?
  - What is your perspective on the comfort and acceptability of NG tube placement for patients? Specifically, how do children generally respond to the procedure, and what measures are taken to ensure their comfort?

12. *What do you think of the use of CAD/AI X-ray in those settings for adults and children?*

Use these prompts as optional tools to aid understanding or provide examples, but adapt or skip them based on the interviewee and interview context

- ➔ *Possible probes*

- Specifically, how do these systems address potential challenges such as the risk of missing non-TB lesions, and what is the impact on patient and healthcare worker trust?
- How well do patients understand that AI, rather than a human, interprets their X-ray images, and how does this perception affect their trust in the diagnosis? - --
- Additionally, how do these systems influence radiologists' practices, and what are the considerations regarding equipment reliability, data ownership, and the security and confidentiality of patient information?

13. *You may be familiar with pooled testing, commonly used during the COVID-19 pandemic. It involves combining samples from multiple individuals with presumptive TB and testing them together. If the pool is positive, each sample is retested individually; if negative, all samples are considered negative. What are your thoughts on using pooled sputum testing in a TB diagnostic algorithm either for screening or diagnosis for adults or for children in those settings?*

g) Adaptability

14. *How transferable or adaptable do you think a combination of tests is? For example, how do you think a combination of test that was approved for PCF would perform during ACF?*

15. *What modifications (i.e. to the tests themselves or implementation/strategy) might be necessary to ensure these tests are applicable in varied contexts beyond primary healthcare settings?*

h) Perceived Effectiveness

16. *Standard of care at primary healthcare level usually relies on W4SS followed by sputum smear microscopy (SSM). How do you think TB test combination will compare to SSM in terms of accuracy, reliability, timeliness, and cost-effectiveness?*

i) Motivation

17. *What evidence would motivate you to advocate the use of combinations of TB tests to health care workers and to health seeking people?*

j) Opportunity/Resources mobilisation

We already talked about the contextual fit, challenges and facilitators for introducing TB test combinations in primary healthcare settings.

18. *Now I would like to ask from your perspective, what challenges and facilitators do you foresee in making the use of TB test combinations sustainable over the long term?*
    - ➔ *Possible probes if needed to support interviewee responses: human resources, supervision, training, procurement, maintenance*

19. *Assuming TB test combinations prove to be cost-effective at primary healthcare level or in specific key or vulnerable populations, what factors and strategies are essential for scaling them up and securing long-term success with strong political commitment from key stakeholders?*
    - ➔ *Possible probes if needed to support interviewee responses: procurement of equipment (X-ray, Xpert machines), human resources in some areas/settings,*
    - ➔ *Possible probes if needed to support interviewee responses: WHO rapid communication + WHO guideline development group endorsement, donors (e.g. Global Fund) recommendations*
    - ➔ *Possible probes if needed to support interviewee responses: show case at global level (UNHLM, Union conference or other international event) and regional level (WHO regional webinar), advocacy from international agencies (STOP TB, World Bank, GF, USAID, CHAI, Damien Foundation, US CDC, IOM, KNCV, ….), political commitment, manufacturers support (Cepheid, AI providers),*

IV. Closing Remarks and Thank You

1. *What is the most important message that you want us to take away from this interview?*
2. *Is there anything else that you would like to add about any of the topics that we've discussed or other areas that we didn't discuss but you think are important?*
3. *Do you have any questions?*

Thank you for your time and participation in this interview. The information that you provided to us will be very helpful in this project. Your responses will be aggregated with those from other participants from other countries. Please feel free to contact us if you would like to receive a copy of the final report about these groups' discussions. Once again thank you for your time and have a nice day/evening.

Topic Guide for Focus Groups Discussions with Healthcare workers (~ up to 120 minutes total)

I. Introduction - Ground rules

Always remember that the participants are the experts, and they have information that you do not have but would like to learn.
Welcome and thank the participants for taking part in the discussion and remind them that they have been asked to participate as their point of view is important.

- *Hello. My name is [lead interviewer's name], and I'll be guiding this discussion with you today. We are also pleased to be joined today by [research assistant/note taker].*
- *First of all, we would like to thank you for agreeing to participate in this focus group discussion. You have been asked to participate because your point of view is crucial for the success of the project we are currently conducting.*
- *The discussion will last about 2 hours.*

A. Consent form and need for consent and signature

- *Have you received the participant information sheet and had a chance to read it? Do you have any questions?*
- *Feel free to ask questions anytime during our conversation.*
- *If there's anything you prefer not to answer, just let me know, and we'll move on*
- *You can stop the interview at any time without needing to explain.*
- *Please remember that we want to know what you think and feel and that there are no right or wrong answers.*

B. Reinforce that the discussion will be anonymous

- *Everything you share will be kept confidential. Your name and organization will not be linked to anything you say and will remain anonymous at all times.*
- *Your responses will be written anonymously and reported in aggregate. No one will know how you, as an individual, responded in the final report.*
- *We'd like to record this session to help with summarizing and transcription. Recordings will be securely stored and destroyed after transcription, and no identifying information will be included in the notes.*
- *Is it OK if I audiotape/videotape this interview today?* **[Turn on recording equipment]**

C. Participant Introductions

Ask each participant to introduce themselves, including if they feel comfortable, their name, occupation, and any relevant affiliations or experiences.

If you agree, could you please introduce yourself and share your role in TB diagnosis and care? How long have you been involved in TB care and treatment?

II. Background

Explain purpose of the interview, that it is meant to assess thoughts, impressions, information and feelings about Start4All tests and test combinations.

Today we will be discussing about your thoughts regarding healthcare service and more particularly tuberculosis services. The objective of our project is to assess the performance of WHO-approved TB tests and test combinations in primary healthcare settings and among key and vulnerable populations, including those living in communities with high HIV prevalence, rural or informal settlements with poor access to health facilities, marginalized groups like refugees and nomads, and children.

By combining TB tests, we mean using tests sequentially to enhance accuracy and cost-effectiveness. The idea is to first use a screening test to identify likely TB cases, then follow up with a confirmatory test. A successful combination should be feasible to deliver, acceptable to users and providers, increase accurate TB diagnoses, exclude non-TB cases, and be cost-effective for the health system.

Use the visual/illustrative handout/summary that was given at the time of the recruitment and re-explain briefly what we mean by combinations of TB tests, giving some concrete examples graphically.

Today's interview focuses on TB test combinations for screening and diagnosis. We want your insights on the main barriers and enablers affecting their acceptability, feasibility, and scalability. This will help us ensure that TB services meet the needs of populations lacking access.

III. Discussion/Questions

- Discuss participants' opinions on the acceptability and also feasibility and scalability of implementing combinations of TB tests within their healthcare settings.
- Explore any potential logistical considerations or implementation strategies.
- Pose open-ended questions to stimulate discussion and gather insights from participants.

Before we begin, I would like to give some information on what a focus group discussion is and how we are going to proceed. As the name suggests, we will be having a discussion as a group, where everyone listens and talk to each other, in order to share thoughts and experiences. Please remember that we want to know what you think and feel and that there are no right or wrong

answers. It is totally fine to have different points of view, and we encourage you to express your opinion and share your own experiences even if they may be different from other people's.

We would like the discussion to be informal. I will lead the discussion by asking questions and then please feel free to discuss amongst yourself and respond directly to the comments made by others. Please let me know if you don't understand any questions. I am here to listen and make sure everyone has a chance to speak and share their thoughts, so please feel free to speak openly and honestly.

There are no judgements made in this room. We also would like to request that none of the information or personal comments made during the discussion are shared outside of this room.

a) Affective attitude - Feelings towards the intervention

1. *What do you think of the idea of combining different TB tests to perform a screening or triage step first and then, for those screening positive, a confirmatory test?*
   - ➔ *Possible probes if needed to support interviewee responses: What could be the benefits/drawbacks?*

b) Capability/Practicality

2. *How easy or complicated would it be to explain the concept of combination of TB tests to people attending primary healthcare facilities and how can these challenges be addressed?*

c) Burden

3. *How do you think different **health care workers** will perceive the use of combined TB testing methods? For example, healthcare workers can include nurses, radiographers, radiologists, laboratory technicians, physicians/doctors, and community health workers.*
   - ➔ *Possible probes using examples listed below if needed to support interviewee responses:*

     - How might nurses involved in TB screening view the implementation of these methods?
     - What could be the perspectives of radiographers who conduct chest X-rays (CXRs) in this context?
     - What challenges or benefits might radiologists see with this approach?
     - How would laboratory technicians working in district-level labs perceive the use of combined TB tests?
     - How might physicians/doctors interpret the use of combined tests in their clinical practice?

- *What are the thoughts of community health workers on implementing these methods?*
- *Foreseen challenges, high workload, limited number of staff, technical capability*

4. *How do you think **people with presumptive TB** will perceive/accept the use of combinations of TB tests?*
   - ➔ *Possible probes if needed to support interviewee responses: stigma, discrimination, time (including waiting time), costs, trust in the test, having to give more than one sample, types of sample*

d) Social influences

5. *What social or cultural factors might influence the acceptance and use of the TB test combinations among healthcare workers? Among health seeking people?*

6. *How can these social or cultural factors be leveraged or mitigated to ensure successful implementation?*

e) Ethicality

7. *All the TB tests are WHO-approved for safety and effectiveness for certain groups of population. Do you have any ethical concerns about using a combination of these tests, such as patient consent, privacy, or potential burden on patients?*

f) Intervention coherence / Contextual Fit

Now I would like to talk about the contextual fit and how appropriate/relevant do you think combinations of TB tests is for primary healthcare settings or for outreach ACF campaigns (in remote hard to reach areas, or overcrowded slums for example).

8. *What are your thoughts on the use of blood tests (e.g., CRP) in these settings? How do finger prick and venipuncture methods compare for use in adults and children? What factors (e.g., lab needs, materials, skillsets) influence the choice of method in these settings?*

9. *What do you think of the practicality and feasibility of collecting urine and the use of urine tests (including LAM) for adults and children in those settings?*
   Use these prompts as optional tools to aid understanding or provide examples but adapt or skip them based on the interviewee and interview context.

- ➔ *Possible probes: Urine Collection Process:*
  - Toilet facilities are available to patients? Are they clean and private?
  - Issues with dehydration affecting sample collection?
  - Are there concerns about urine leakage due to improper sealing?

- ➔ *Possible probes: Challenges in Urine Collection:*
  - Do patients struggle with directing urine into containers, especially with limited water or toilet paper?
  - How does timing and the time required to provide a sample affect the process?
  - What aids could improve the collection process?
  - What other challenges have been encountered with other urine-based tests like pregnancy or Urinary Tract Infection (UTI) testing?

10. *What do you think of the idea of collecting stools samples for adults and children in those settings?*
    Use these prompts as optional tools to aid understanding or provide examples but adapt or skip them based on the interviewee and interview context.
    - ➔ *Possible probes - Sample Production:*
      - Can individuals produce stool samples on demand, or would multiple clinic visits be needed?
      - Handling and Storage: How feasible is it for patients, especially parents, to handle and store stool samples properly before bringing them to the clinic?
      - Comfort and Privacy: How can clinics ensure comfort and privacy during the collection process?
      - Parental Involvement: For children, would stool collection require multiple visits or significant parental involvement? Are guardians comfortable managing and transporting stool samples?

11. *What do you think of the idea of collecting gastric aspirates samples in those settings, especially for children?*

    Use these prompts as optional tools to aid understanding or provide examples but adapt or skip them based on the interviewee and interview context.

    - ➔ *Possible probes*
      - In your experience, is it typically necessary for patients to be admitted to the hospital for gastric aspirate sampling, or can this procedure be conducted in an outpatient setting?
      - How common is it for healthcare professionals to be trained in placing nasogastric (NG) tubes?
      - What is your perspective on the comfort and acceptability of NG tube placement for patients? Specifically, how do children generally respond to the procedure, and what measures are taken to ensure their comfort?

12. What do you think of the use of CAD/AI X-ray in those settings for adults and children?

Use these prompts as optional tools to aid understanding or provide examples, but adapt or skip them based on the interviewee and interview context

- ➔ *Possible probes*
  - Specifically, how do these systems address potential challenges such as the risk of missing non-TB lesions, and what is the impact on patient and healthcare worker trust?
  - How well do patients understand that AI, rather than a human, interprets their X-ray images, and how does this perception affect their trust in the diagnosis? - --
  - Additionally, how do these systems influence radiologists' practices, and what are the considerations regarding equipment reliability, data ownership, and the security and confidentiality of patient information?

13. You may be familiar with pooled testing, commonly used during the COVID-19 pandemic. It involves combining samples from multiple individuals with presumptive TB and testing them together. If the pool is positive, each sample is retested individually; if negative, all samples are considered negative. What are your thoughts on using pooled sputum testing in a TB diagnostic algorithm either for screening or diagnosis for adults or for children in those settings?

g) Adaptability

14. How transferable or adaptable do you think a combination of tests is? For example, how do you think a combination of test that was approved for PCF would perform during ACF?

15. What modifications (i.e. to the tests themselves or implementation/strategy) might be necessary to ensure these tests are applicable in varied contexts beyond primary healthcare settings?

h) Perceived Effectiveness

16. Standard of care at primary healthcare level usually relies on W4SS followed by sputum smear microscopy (SSM). How do you think TB test combination will compare to SSM in terms of accuracy, reliability, timeliness, and cost-effectiveness?

i) Motivation

17. What evidence would motivate you to advocate the use of combinations of TB tests to health care workers and to health seeking people?

j) Opportunity/Resources mobilisation

We already talked about the contextual fit, challenges and facilitators for introducing TB test combinations in primary healthcare settings.

18. *Now I would like to ask from your perspective, what challenges and facilitators do you foresee in making the use of TB test combinations sustainable over the long term?*
    - ➔ *Possible probes if needed to support interviewee responses: human resources, supervision, training, procurement, maintenance*

19. *Assuming TB test combinations prove to be cost-effective at primary healthcare level or in specific key or vulnerable populations, what factors and strategies are essential for scaling them up and securing long-term success with strong political commitment from key stakeholders?*
    - ➔ *Possible probes if needed to support interviewee responses: procurement of equipment (X-ray, Xpert machines), human resources in some areas/settings,*
    - ➔ *Possible probes if needed to support interviewee responses: WHO rapid communication + WHO guideline development group endorsement, donors (e.g. Global Fund) recommendations*
    - ➔ *Possible probes if needed to support interviewee responses: show case at global level (UNHLM, Union conference or other international event) and regional level (WHO regional webinar), advocacy from international agencies (STOP TB, World Bank, GF, USAID, CHAI, Damien Foundation, US CDC, IOM, KNCV, ….), political commitment, manufacturers support (Cepheid, AI providers),*

I. Closing Remarks and Thank You

1. *What is the most important message that you want us to take away from this interview?*
2. *Is there anything else that you would like to add about any of the topics that we've discussed or other areas that we didn't discuss but you think are important?*
3. *Do you have any questions?*

Thank you for your time and participation in this interview. The information that you provided to us will be very helpful in this project. Your responses will be aggregated with those from other participants from other countries. Please feel free to contact us if you would like to receive a copy of the final report about these groups' discussions. Once again thank you for your time and have a nice day/evening.

Topic Guide for Focus Groups Discussions with people with TB (~ up to 120 minutes total)

I. Introduction - Ground rules

Always remember that the participants are the experts, and they have information that you do not have but would like to learn.
Welcome and thank the participants for taking part in the discussion and remind them that they have been asked to participate as their point of view is important.

- *Hello. My name is [lead interviewer's name], and I'll be guiding this discussion with you today. We are also pleased to be joined today by [research assistant/note taker].*
- *First of all, we would like to thank you for agreeing to participate in this focus group discussion. You have been asked to participate because your point of view is crucial for the success of the project we are currently conducting.*
- *The discussion will last about 2 hours.*

A. Consent form and need for consent and signature

- *Have you received the participant information sheet and had a chance to read it? Do you have any questions?*
- *Feel free to ask questions anytime during our conversation.*
- *If there's anything you prefer not to answer, just let me know, and we'll move on*
- *You can stop the interview at any time without needing to explain.*
- *Please remember that we want to know what you think and feel and that there are no right or wrong answers.*

B. Reinforce that the discussion will be anonymous

- *Everything you share will be kept confidential. Your name will not be linked to anything you say and will remain anonymous at all times.*
- *Your responses will be written anonymously and reported in aggregate. No one will know how you, as an individual, responded in the final report.*
- *We'd like to record this session to help with summarizing and transcription. Recordings will be securely stored and destroyed after transcription, and no identifying information will be included in the notes.*
- *Is it OK if I audiotape/videotape this interview today?* **[Turn on recording equipment]**

C. Participant Introductions

Ask each participant to introduce themselves, including if they feel comfortable, their name, occupation.

II. Background

Explain purpose of the discussion, that it is meant to assess thoughts, impressions, information and feelings about Start4All tests and test combinations.

In the project we are conducting, we are evaluating a new approach for TB diagnosis that we call "combinations of TB tests" for TB diagnosis in primary health care settings and for key and vulnerable populations, including those living in communities with high HIV prevalence, rural or informal settlements with poor access to health facilities, marginalized groups like refugees and nomads, and children.

By combination of tests, we mean that we are going to first use some tests that will give us some indication on the possibility of an individual of having TB. If this test is positive, then we are going to use a second test to confirm whether that individual has TB or not. If the first test is negative, we will consider the individual is unlikely to have TB, and the second test will not be offered. All tests evaluated in our project are WHO-approved.

Use the visual/illustrative handout/summary that was given at the time of the recruitment and re-explain briefly what we mean by combinations of tests, giving some concrete examples graphically.

Today we will be discussing about your thoughts with regards to healthcare service and more particularly tuberculosis services and the idea of combination of TB tests. This information will be very helpful to us so we can make sure we meet expectations and needs for people to access TB services.

III. Discussion/Questions

- Discuss participants' opinions on their perceptions about combinations of TB tests.
- Pose open-ended questions to stimulate discussion and gather insights from participants

Before we begin, I would like to give a few information on what a focus group discussion is and how we are going to proceed. As the name suggests, we will be having a discussion as a group, where everyone listens and talks to each other, in order to share thoughts and experiences. Please remember that we want to know what you think and feel and that there are no right or wrong answers. It is totally fine to have different points of view and we encourage you to express your opinion even if it is different from the other's.

We would like the discussion to be informal. I will lead the discussion by asking questions and then please feel free to discuss amongst yourself and respond directly to the comments made by others. Please let me know if you don't understand any questions. I am here to listen and make sure everyone has a chance to speak and share their thoughts, so please feel free to speak openly and honestly.

There are no judgements made in this room. We also would like to request that none of the information or personal comments made during the discussion are shared outside of this room.

A. Acceptability

a) Affective attitude - Feelings towards the intervention

1. *First, I would like to start by asking you what is your community's perception about TB disease?*
   - ➔ *Possible probes: how are people with TB perceived, social and professional impact for people with TB*

2. *What do you think of the idea of going through several tests and possibly collecting several types of samples to be diagnosed with TB?*

3. *What would need to be the performance of such tests to make you choose a combination of a single test?*
   - ➔ *Possible probes: more likely to pick up TB when I have it, more likely to exclude TB when I don't have TB, cheap, rapid, type (i.e. non-sputum vs sputum)*

b) Burden

4. *How would you feel if the doctor asked you to provide several types of samples for TB diagnosis, such as:*
   - o *Sputum for adults? For children?*
   - o *Blood for adults? For children?*
   - o *Urine for adults? For children?*
   - o *Stool for adults? For children?*
   - o *Gastric aspirate for adults? For children?*

5. *Which sample would you feel more comfortable to provide among the samples we mentioned?*
   - *Do you have any reasons for selecting this sample?*

6. *Which sample would you feel **not** comfortable to provide?*
   - *Do you have any reasons for selecting this sample?*

7. *Are there any other samples not mentioned that you think would be more comfortable to provide?*

8. *What is the maximum number of different samples you would feel comfortable to provide for TB diagnosis including a potential initial screening test?*

- ➔ *Possible probes: Would your comfort level change if all the tests could be completed within a short time frame, such as within an hour or a single day?*

9. *How did you feel about being asked to get a chest X-ray for an adult? For a child?*

10. *What would you feel like if that X-ray was read by a computer programme (AI) rather than a human being? Why would you feel that way?*

11. *In some places, health services are trying a method called 'pooled testing.' This means that instead of testing each person's sample separately, they mix small parts of samples from a few people and test them together. If the group test is negative, everyone in the group is considered TB-free. If it's positive, they go back and test each person's sample to see who has TB. How would you feel if your sample was tested this way?*

c) Ethicality

12. *Thinking about your recent illness with TB and journey to seek diagnosis, medicines, and care, I would like to hear about your experience at the health facility and ask how the quality of the service you received was.*
    - *How did you feel about coming to the health facility?*
    - *Could you please describe the health care workers behaviours and how you were welcome and treated?*
    - ➔ *Possible probes: behaviours, respect, politeness, curtesy, friendliness, professionalism*

13. *Did the health facility offer support for any additional concerns you might have had, like transportation, financial difficulties, other medical care needs, or substance abuse? If they didn't, would you have wanted help with these or other issues?*

14. *When you visited the health facility, doctor, or another health worker, was there anything you didn't like or understand?*

d) Intervention coherence

Use the visual/illustrative handout/summary that was given at the time of the recruitment to show people examples of test combinations as illustrations and consider someone's potential journey through those. Be cautious not to persuade/influence them and make them think only about certain TB test combinations.

I now would like to have your opinion on the potential journey an individual may need to go through if we were to use combinations of tests for TB diagnosis. We are not saying that this

combination will be used as the routine standard of care, but this is more for you to visualise what this strategy would imply by using a concrete example.

So let's assume you attend the health facility with symptoms of TB. The doctor asks you to provide one blood sample and one urine sample in order to perform some preliminary tests. And then he asks you to wait for one or two hours, or to come back in the afternoon. Then in the afternoon, the test result comes back ***positive****, and the doctor now asks for a sputum specimen in order to perform a TB diagnosis test. And he asks you to come back the day after to get your result.*

15. *How would you feel if you were to go through this journey?*

A second scenario. You present at the health facility with symptoms of TB. The doctor asks you to provide one blood sample and one urine sample in order to perform some preliminary tests. And then he asks you to wait for one or two hours, or to come back in the afternoon. Then in the afternoon, the test result comes back ***negative****, and the doctor says you are unlikely to have TB and therefore there is no need to test you for TB. But you yourself believe you may have TB.*

16. *How would that make you feel?*

17. *What would be your ideal turnaround time between sample collection and final diagnosis results feedback?*

e) Opportunity costs

18. *Now I would like to talk about the costs you had to face when seeking healthcare for your symptoms related to TB. Medical and non-medical costs can include expenses such as transportation, accommodation, food, and additional tests, as well as indirect costs like lost income or time.*
    - *Can you describe the major costs you encountered while seeking healthcare for your TB-related symptoms? Why were these significant for you?*
    - *Were there any other costs or challenges you faced that are not mentioned here?*
    - *Will you receive reimbursement for any of these expenses?*
    - *Do you receive any incentives for screening or testing as part of a government scheme? If so, what kind?*
19. *Now I would like to talk about the impact once you and/or others noticed you were unwell, and you decided to seek diagnosis, medicines, and care from the health system.*
    - *How did taking time off work to seek care affect you? What challenges did you face during this time?*
    - *Can you describe any financial difficulties you experienced related to healthcare costs? How did these difficulties impact you or your family?*

- *Did you need to borrow money or find other financial support to cover these expenses? How did this affect your overall financial situation?*
- *How did you feel psychologically and mentally about the possibility of being diagnosed with TB?* (anxiety and stress, risk of social stigma/discrimination/judgments)

f) Perceived Effectiveness

20. *What factors would make you prefer combination TB tests, and what concerns or challenges might make you hesitant to use them in terms of how well they would perform?*
21. *How would it influence your decisions to seek care?*

g) Self-efficacy

22. *I would like to ask you about the community's awareness about health-related issues and availability of health services*
    - *If combinations of TB tests were available at your health facility, would people from the community be more willing to come and get tested at the health facility?*
    - *What needs to be done so that community/people with presumptive TB would be supportive of such TB test combinations being standard of care and willingly access and complete them?*

IV. Closing Remarks

- Summarize key points discussed during the focus group discussion.
- Thank participants for their valuable insights and contributions.
- Provide information on follow-up steps or next stages of Start4All

1. *What is the most important message that you want us to take away from this interview?*
2. *Is there anything else that you would like to add about any of the topics that we've discussed or other areas that we didn't discuss but you think are important?*
3. *Do you have any questions?*

Thank you for your time and participation in this interview. The information that you provided to us will be very helpful in this project. Your responses will be aggregated with those from other participants from other countries. Please feel free to contact us if you would like to receive a copy of the final report about these groups' discussions. Once again thank you for your time and have a nice day/evening.

Pooled testing quotes

- When the pooling test system is fully implemented in the future, costs will definitely decrease.

- Ok, as far as I'm concerned, this pooled testing method is really beneficial, in the sense that we would have saved time and, economically speaking, we would have wasted fewer cartridges. With a small number of cartridges, we can test many patients at the same time.

- For my part, I find that, as he said, but there's a bit of nuance. Why do I say that? Because I was in a situation last year, this issue of pooled testing. We had declared a multi-resistant. Now I was obliged as the team sent me into the community to look for the guy to collect a sample. Seeing as it was a positive resistant, they tested as an individual. They didn't see anything. Now I had to go down to the community, which was so far away, I was even sick. I recovered the guy, I collected a sample and sent it for testing. They did it and it came back negative. They asked me to go back again, I made three rounds and he provided good sputum. At this level...and they followed me with the same problem, not even later than Monday. I was asked about his welfare, and I've always gave a report about the guy, that he's neither positive-sensitive, nor resistant. At this level, it frightens me a little. Pooled testing in question. Yes, it's all well and good, cartridges are saved, we save time, but at a certain level...

- Personally, I think it will save on resources because if you pool multiple patients together, let us say five together, then you pool with one cartridge, then if it is positive then you will have to do individuals. So, if it is negative, then you don't...

- That one will also, because you know with TB the possibility rate it is so small. So, we have, the negatives are so many. So, those ones will also, when you pool them together, then you will save on the cartridges and of course the workload.

- I think the pool is a good one. It is cost effective considering the cost of cartridge especially when we go for active case finding and between the individual assessments for them, it will cost more. Like now we are hearing no cartridge in the country, so pooling samples will be effective. Positive ones will be checked. However, I think we should not just rely on, I think it's best to use, just like combination tests, to use other platforms also, because there could be possibility of missing a positive sampling in pool I suspect So, such that, as we use pool when we detect a positive, then we do the individuals. Then if we also get a negative pool, and we detect any positive on all that testing platform, we should also do an individual for each of those, for that particular pool that is negative. So, that’s just it.

- I think we shouldn’t just rely on the pooling aspect, I think individual testing should be done because I’ve encountered a situation whereby a sample when it was run individually, it showed very low TB and when it was pooled together, it came out negative. So I think they shouldn’t just rely on the pool sampling.

- I think there are grades that are NTB. So for those I experienced that as some, there was a particular one, this particular individual, I think, you know, maybe about two of them in a pool, their individuals were negative and I think they were once positive for TB. They've treated TB years ago. So now these two individuals, the X ray was abnormal, it showed that they had abnormal. However, their individuals were negative. But when they were that particular, those samples were put together, it showed positive. So maybe their immune was low so by the time we brought them together it became positive. So also for the individual that was very low, like she said by the time you bring them together, it mixes with other cases but if we had done only the pool, it would have said negative. So we won't have the chance of now doing the individual that was there, we will have a very low trace.

- Why are we still doing this pooling? Is it because of cost? I think we should not be talking of cost at this stage where we are, seriously speaking, they will get to a stage where we have reached as far as elimination in the general population is concerned. Then at that stage, we can be talking about pooling but now that we want to drastically bring down MTB in the pool, in the population pool. My personal opinion is that we should not be talking about the pooling test for now, let's actively get these people and treat them. That's what I feel.

- You're asking about that one test for treatment.Yes, clinical judgment is needed. If a repeat test is needed, we do it, maybe keeping a two-week follow-up. That follow-up patient gets caught the next time. And next time, I won't include them in pooling. I'll test them individually. Then the chance of missing cases will certainly be lower.

- This is a complex question requiring detailed consideration. The work depends on various factors - if a doctor has dedication and care for their work, they shouldn't lose enthusiasm. There's no reason for diminished enthusiasm. As I understand this method for our resource-limited country, these patients don't have TB. I understand they don't have TB, but verbal confirmation isn't sufficient. Many such cases present daily. We conducted pooling with them, performed tests, and I know from history it can be determined this isn't TB. We can at least utilize this approach in those cases. This method you're seeking to establish is essential for this component. Therefore, executing this necessary work shouldn't cause irritation or loss of enthusiasm. However, individual variations may occur.

- For screening, I understand that it is interesting. Because you end up having a larger arm to reach the population suspected of having the disease, or rather, potentially

exposed to the disease, and you use resources. So I think you have to consider issues that go beyond my perspective on the methodology itself. Depending on the tests, the tests present a molecular key, but that is not the manufacturer's recommendation. Therefore, we may have false results or discordant results because the methodology is not followed according to the manufacturer's recommendation. In addition, there is a reason for it being recommended that way. No matter how varied they are. However, for screening, mass population and everything else, I tend to think that this is something that helps. However, all the initiatives that we have seen thus far are not promising from the point of view of the outcome of the patient's treatment. For example, because many that I saw, that I followed, there were many that did a pool of samples, grouped samples. They put them in the test, they came back positive, and you had to chase after the five, for example, to treat them individually. Therefore, if you have easy access to the population, that is great. However, if not, you add some extra time. In addition, then, when all is said and done, will it add up? Therefore, I think it is worth studying a little about what the protocol will be like and the conditions under which the study will take place. For the diagnosis itself, the person who comes in with all the symptoms, the stomatology of the disease, will take the test. I do not think it is worth waiting to collect more samples to run a test. Therefore, these people already need to have an individualized test. Therefore, that is why I think there has to be a distinction, whether the proposal is for biometrics, for diagnosis, and then, it is possible to provide better guidance.

- So I think a test that is expensive. So I think it's a welcome strategy. But, again, it requires a lot of organizational capacity in the laboratory, which is not small, right? You have to have a very attentive professional who pays attention to the details, right? You have to collect several samples. Then you can separate which samples are which, which were in that pool, right? For unloading, so you can repeat the test.

- And then, well, I want to believe that the X-rays that we'll be using to screen people, they have been designed in a way that the irradiation has…they will not irradiate a lot, because you want to look at maybe the disadvantages of doing X-rays. We don't know how I have been informed that they could be safe for pregnant women. If you want to bring in X-rays to use systematically for children, I mean, to diagnose TB. Are they safe for the pregnant women? What's the dose of radiation that goes in those equipment, those are unknowns that maybe the manufacturers will give more information on whether we can use to diagnose pregnant women and to screen, or only children, you know. But I think the multiple methods may not have a lot of disadvantage, but what we know is that it could help us to…we need an initial investment. And it can also, well, no, no, I think being economical or cost-effective is an advantage of… Yeah, because if you even propose pooled testing, might be cost-effective or more economical, less costly.

- In Primary healthcare settings. Yeah, pooled testing is a method that should be encouraged in low-income settings like ours, where we are always having stock out or things like that. Because it's been proven to be economical, use less to test more. So, it would be good to do it, to screen and especially you're doing pool because you're doing maybe a molecular test that is very, very sensitive. So, you pool, it helps you to economize on the use of reagents and you pool and only the positive pools will be offered individual testing. So, it's good. It's true that you might say that it takes more time to pool the samples together, you know. But in the end, it's economical and it takes less time because if you pool three and test and they're negative, that's it. You move to the next. And by the end of the day, you might do less work, even though apparently it might look like you're spending more time to prepare your pools.

- Well, pooling, I think, has already been validated by the program. It's been validated and shown to be effective. When the target is well respected, there's no problem. Now, the problem we encounter in the field is that some people do it just to get it out of the way. When the examination forms are not properly filled in, it causes a problem. Because in pooling, for example, re-treatment cases cannot be included. In-patients can't be included, children under the age of 5...there is a particular target. Because when there's a good chance of diagnosis....so, when you pool, you're going to lose cartridges in that target. Right. But if you respect the target, it's really, it's really beneficial. Because it saves cartridges. Also taking into account that we lose a lot of cartridges. But now, as I say, that the target should really be respected. And now, I think we need to continue working on practitioners, especially laboratory technicians. Laboratory technicians and prescribers...prescribers in filling-in the examination request form correctly. Because there is an examination request form for the Program. Because sometimes, a practitioner, a consultant...he doesn't use the form, he sends it to the lab. Okay. At the time, the laboratory technician doesn't know if it's a person living with HIV, if it's a hospitalized patient, if it's a case of re-treatment. He doesn't know. And now he does it, and it ruins everything.

- Personally, I think lot of challenges with logistics. We are having a lot of challenges with our cartridges, reagents, stuff like that. But still, in that hall, we had some stakeholders who were still not satisfied with it, because of the possibility of cross-contamination. During the manipulation of the different samples, we could contaminate another sample. Let's say, for instance, we have two cups here, and maybe the technician is crossing and pulls there and forgets to change the pipette, and later collects from the next cup. And if this was a positive, automatically, this one gets contaminated as well. So, some other colleagues had a little bit of conflict, but I feel that if it is done properly, according to the way it's supposed to be done, I think it's a good idea. It's a good idea, implementing the pooled testing.

- It's challenging in the sense that we know our laboratory settings have a basic standard of setup. I cannot just get up this morning and transform my office or my bedroom to a laboratory. There are some certain standards I have to meet to be able. So, if we're at the level of the community, if we want to do pooled testing at the community level, I think there are a lot of things that need to be taken into consideration. How can I call it? Mobile laboratory. Some kind of a thing that when you get to the… You see, usually in this, when we have a football match or whatever, there's usually this portable public toilet that is portable. That they take to the field, after that they can… So, if you have a structure that is easily mountable, that can easily be mounted, and that has a little bit of restriction to minimize air flow, circulation, dust from outside getting into our samples. I think it would be a good idea. So, starting with that, the setting, yeah. And then now you have to make it in such a way as well. I don't know how the pooled testing works. I've never seen it physically, but I think the size of the equipment and so on and so forth, that is supposed to be small enough, portable enough. Because as I say, in the XXX XXX, we don't have roads. It's really challenging.

- I think pooled testing is very cost effective. Yes. We've done it at our facility in XXX. I remember there were times that we could not really do the tests easily because getting the cartridges was a problem. But with the pooled testing, we get so many samples, small-small from many samples and then run together. If it's negative, then okay, all of them are negative. We only go now to test one after the other if we have a positive result so, I think that helps to save a lot of cartridges for use. So, I think it's a great idea when I heard about it.

- You save time that would really help us. No, I'm saying…no, I'm saying pooling, I'm very prompt. In other words, if pooling can give me the results in less than 24 hours, why not? You save on samples, you save on time, you save on everything. In other words, you mix three or four sputum samples, as you said, and test them. If it's negative, everyone's negative and we've all won, haven't we? We've also saved time, haven't we? I think that I'd emphasize more on the people doing the work, eh, who's going to do that? Because if you…it's all good eh, it's all very good eh, on paper, as we're discussing here and everything, that's fine. But afterwards, will you have competent personnel? Well, you need qualified, competent personnel who want to do what they're doing. Because we all know that caring for a patient really requires passion and all that. So, if it's like, ah, now I have ten over there…you're talking about all… pooled testing. That's not my fear. When you have to go away, will you leave it to the DTC? Maybe you'll say that okay, we do not accept, maybe…I don't know, eh…we don't accept, maybe 4 or 5 samples. Wouldn't they sit down later and take 20 samples and mix them? That's the thing you have to consider. So, all this boils down to good training. You have to train your staff well. Because we know what happens afterwards. 'I'm even tired today' and all that. Maybe it's because their incentives are late. We know how the country works and everything. After that, they take even six or…'how many samples are even there?' 'A hundred.' 'Just mix the hundred there

let's see'. I can assure you that sometimes I've found myself with TB LAMP, AFB negative sputum of which I had my doubts. Where I had doubts. And I had even expressed my doubts to the director once. I told her that I didn't understand. I mean, you see a sick person, you see everything. It's true that there's also the quality of the sputum. Because sometimes, some patients don't provide sputum. They give saliva. You know what I mean. But now, when it's good sputum and all that, and really, sometimes, we have our doubts.

- I'm wondering why you're pooling samples. Are you trying to increase the bacterial load? Like why? Let me go back to my initial high HIV burden countries. So the reason we use LAM in people living with HIV, especially those with advanced HIV disease and children, is that there is just so much going on. It's hard to diagnose TB with sputum alone. So, if you have a region with a large number of these people, and you dilute the sputum, it seems like we're going backwards. It might work in certain regions, but I can't speak for all of them. I think, yes, there are areas where the disease burden is high, but you don't want people to be diagnosed too late. If people are diagnosed early, I doubt the bacteria would have replicated enough to survive any dilution. So, I'm just not sure pooling is the best approach. I think if we have better screening tools, and then we test only those who need it, we could save resources that way, rather than pooling. Because sometimes we also have rejections on ultra when the sputum was of poor quality. So if we're now diluting with others with poor quality, some with good quality, what happens to the entire pool? Do we now have an error for that entire pool? We could end up with an error for the whole pool, whereas we could have just had an error for the single bad sample. There are other specific challenges. For example, even for the person testing at the Xpert site, instead of just taking one milliliter of sputum and testing it, now I would have to take one milliliter from each sample. There's also the risk of cross-contamination. You can't use the same syringe for all of them; you have to use a different extraction tool for each. If you take a sample here and put it there, when you go back to test them individually, it could be contaminated. So, from a process perspective, it sounds like a nightmare. And also, all of those be contextualized. So yes, that concept in general makes sense to me. I think my hesitancy is always that we are at this point because some of the tools that we're using, which are considered gold standards, are just too expensive. When you put up the pooling and the Ultra pooling example for GeneXpert, I think we—and you can correct me if I'm wrong—only get there because GeneXpert is too expensive. I mean, if it was an affordable test, we would just say, "Test everyone with GeneXpert." It's hard for children, but if we had other sampling techniques, maybe it would still be the gold standard. Sometimes I feel like it's "Why should I learn something new?". So that's always a thing that has to be managed. Change management is really critical. In this case, it's in the way that we communicate what the benefit is. I think starting out with the benefit is always important. The benefit is like we get more accurate tests. We are likely to diagnose the right people at the right time, and so that's why this is

of benefit, as opposed to going into what we're trying to do with the test and then telling them the benefit. So change management, I think, is really critical for these kinds of interventions because people are so used to doing things one way. For GeneXpert pooling, for example, of all the tests that you are doing, it seems to me like the hardest to sell because it's not an individual test. It's like we're going to put this in a pool, and if this pool gets positive, then we will test the whole pool. From a cost-effective perspective, it makes all the sense in the world that you would say, “Why should we test all these samples for GeneXpert, which costs $8 a cartridge? Why don't we just test the ones that we think?” But I don't know if that lands well from the patient's perspective, what they think about that, because it's not like they could be tested or not. That depends on if the batch is tested. Compared to the four others, this is something that would need to be communicated more clearly: what the benefit would be. The rest are individual tests that would be done on an individual as opposed to the GeneXpert pooling. Change management would be really critical. Again, I go back to this: our tests are tests that people have been doing, whether this person is specifically trained as a TB focal person or a healthcare provider in general. They can do this because they've had to interact with CRP and do a sputum test at some point. I don't think they would be really hesitant about these tests. That’s the benefit of this study: it's not something out of the sky; it's things that are already there. We’re just saying if we combine this and this, we get to this, and we get faster at treating the people that we want to treat. So, I don't foresee that it will be a bit of a challenge to convince healthcare providers that they can do this combination of tests because they're not new to them.

- For me, GeneXpert pooling, TB LAM, and chest X-ray can all be feasible at the primary healthcare level. Now, with CRP, whether it requires a phlebotomist or can be done with a finger prick, that’s where I see a potential challenge.
  If a phlebotomist is required and could become a bottleneck. For TB LAM, I don’t see any issue. Providing urine at the primary healthcare level is very feasible. Similarly, with GeneXpert pooling, the patient provides sputum, and the samples are pooled. This doesn’t necessarily require specialized expertise at the primary healthcare level. So, for me, the main challenge is about how you communicate that. Honestly, we would love to move away from smear microscopy. It’s one of the things that really makes me sad about TB. It’s 2024, and we’re still using smear microscopy. It’s really sad. Hopefully, studies like yours will help us move in a different direction. But we do know the biggest bottleneck is the cost. The cost of GeneXpert and molecular diagnostics needs to be at a level that is sustainable for scale-up. As it stands, we can't sustainably scale up GeneXpert. We can't afford to test everyone, and the momentum we're seeing now with TB notification is great. We were having a discussion about this with the team, and we believe that the scale at which we are moving is unsustainable.

- We can trace the history of algorithms back to about 20 years ago, when patients were required to provide three samples each. Why three samples? It was because we discovered that some samples were more concentrated than others. So, the first thing to understand is that regardless of whether we're pooling samples, we need to know which types of samples are more likely to be positive. If I pool samples that are well collected (not saliva-based) and they come out positive, I don't need to keep bothering the patient. I can pool samples from a specific neighborhood and, within two hours, determine which patient is sick. Then, I can say, "You are going to receive treatment," and start counseling right away. The key here is how you communicate this.

- I suppose if it's explained to me. However, it could be quite stressful. For example, if the pool test is positive, and the other three individuals don't have TB, it means only one person out of the four might be positive. But the stress comes from the fact that a positive pool result could make everyone worry. As long as people understand that the positive result doesn't necessarily mean they have TB, just that they could potentially have it, it should be fine. It's important to consider how

- For pooling, my main concern is from an ethical point of view. As you said, we have four samples and test them. If one is positive, then we stress three people by asking them to be tested again, saying, 'You probably have TB, or maybe not.' In this way, you cause stress for the patients. It's one thing if I give my sputum and get a positive or negative result. But if my sputum was positive, I don't know if I was the positive one, or if it was the other person tested with me. Asking someone who is negative to be tested again creates unnecessary stress for them.

- In Korea, we used pooling during the COVID-19 pandemic. If many patients are in an area, pooling can save resources. If one sample is positive, individuals are tested. In areas with many COVID-19 cases, pooling is needed. If all samples test positive, individual testing follows. If many patients are present, individual testing is more economical.

- Yes, pooling works well where there are fewer patients. If there is enough space and equipment, it is feasible in primary healthcare. If there is enough space and refrigeration for samples, it's feasible. Equipment like expert machines and cartridges are needed. If there is enough space for samples, it's possible. I think individual tests and pooling can both be done in primary healthcare if infrastructure is available. In areas with low TB rates, pooling is economical. Pooling works well in areas with a high rate of TB, where most tests are positive.

- Yeah, it will also increase my positive rate. And what it is going to do because in this Start4All, we are bringing in the pooling. It is going to be cost-effective at the end of the day, a good example, if I can give a good example where if I can run 200 samples and I pool those 200 samples, I will only use 50 cartridges instead of using 200

cartridges. And not all those 200 cartridges which are used, they will all give me positive; I will even get 10 positives; and the 10 positives, I will not get them in all the cartridges. Let us say if I get the 10 positives, I have now like 10 cartridges which I am supposed to repeat a 40-sample. So, if I run those 40-sample individual, if I add with my 50 cartridges, I have 90 cartridges, I have 90 cartridges. So, I have saved almost 110 cartridges. So, at the end of the day, it is going to be cost-effective. We don't know what tomorrow will entail, the donors may pull out, will our patients who have been diagnosed with TB, will they really pay 5000 shillings for the test? They won't pay. But if we can bring this, now we can pool our patients together where we have four patients running for the TB which each can only pay 1000 shillings for the test and that one at the end of the day, it will be cost-effective for them.

- Now, for the TB pooling, my thought is that I support it fully, because at the end of the day, it is going to be a cost effective for us on the TB test within our community. So, it is a better idea for us.

- As per now the evidence which can make me to spread this gospel to the community by adopting this combination, is simply, first of all it is just the cost-effectiveness of this combination because most of the time our facilities, we run out of stock of the cartridge; not that we can't afford them; yes, we can afford them but they are expensive. But if you can bring for example this our method of combination where we are also now going to practice pooling, yeah, it is going to reduce our cost; and also it is going to reduce our out-of-stock within our facility.

- The pool is where you were taking for samples for how many people? And you collect… And then… You pick from all of them. And then put in a common cartridge. So, if it is negative, you assume all are negative. If it is positive, now again, you go now for individuals. This pool we were using it at what level? At the level of molecular? We don't wait for the results for the microscopy before we do the pooling? Now, let me answer this thing with a question. We have sample A, B, C and D; their microscopy are collected and they have gone to microscopy. But again, you collected A, B, C and D. Again, you have taken to the pool. And at the pool, they read NEG. If you come back to the individuals that were done for microscopy, and you get one did MTB. And this MTB, being that it is non-mycobacteria, it was not able to pick at the molecular point. What do you do if you had already discharge the rest? So, in a pool, if the pool is negative, will they still go for culture. So, it will be available either way. But if the pool comes positive, then now they do individual, expert again. So now ask me my question.

- The pool is good. At one point, pool is 50-50. It can save the cartridge; it can waste the cartridge. It can save the time. It can extend the time. It depends with the scenario. If the Pool is negative, we have saved, and we have not also wasted time. Now if pool is positive, we again have to go for another hour. As we look for

individuals. So okay, the pool, it is either way. I don't know where to place it. Okay. If most turn out negative, it is good for us. So, we save a lot of cartridges. But it is it is something that is worth trying. Because with the experience, we have realized our positivity is also low. We are not achieving, it means a number of our samples that we submit are negative. So in that case if we could employ the pool, then we would have saved a number of cartridges. And also saved time. Because in pooling, it is like you can run 16 samples at a go. Four in one, and then one-one-one, right?

- I feel like it's cost effective because there wouldn't be a need to use more cartridges once a pool turns out negative. It's cost effective, it's also reduced, it's not time consuming also. It reduces on the time used to run these samples.

- For primary health care, we can, we may neglect the pooled system. But for the outreach, the pooled system can work, just so that we test them all at once; if they turn positive, then we take the initiative of now testing individually. Remember here we are just getting subjects; we may have a very large number, so maybe we can subdivide them into, if we are having a hundred participants, we can give them maybe 30, 30, 30, you see, then we test them as a pool. So, you can get that one pool is the only one that turned positive. So, in that pool of 30, we now decide to test them individually.

- So that one depends on the associated risk of the environment that we are in. Okay. Then pull it and work; so that we do four, four, four, four. So, in this four, if one pool turns positive, then we test all the individuals. But if it is negative Then you discard all. Yeah, that one can be save us some time.

- So, the pooling system, I do have a little bit of reservation of that person but it may has got advantages and disadvantages. The advantage is that: you are using few testing devices for a number of samples at once. So, for example you use one cartridge to test maybe five samples instead of using five cartridges for five samples. However, we have to consider two things; is the test we are using a molecular test, because why I am asking that question is that: when you pool samples, one: you may affect the dilution factor of the sample or concentration of the pathogens which you are looking for. So if it is not a molecular test which can pick the DN of even a single bacteria and you are using a different test which cannot pick that but can just read the number of bacteria in that sample. Then you have to think of dilution factors. So that you don’t miss a positive sample. But if you are using a molecular test you know even if there is one bacteria, it will still pick the DNA and amplify it, so that you know that they are in one of the samples of this bacteria present. So those are some of things you may need to consider.

- So this is something that was used like with COVID during the COVID time where they'll pool samples due to the way and also minimizing testing reagents. The challenge is no, you know in the facility we have infrastructure already there in part

is there, there needs healthcare worker is there we collect sample we can maintain the first initial sample and then we pool them together. But in the community setting now there are a lot of things that need to be in place.

- I think the key issue that I may have, especially with combining the tests is: I think I have said it that for one to obtain a multiple samples from the same patient, sometimes it can be uncomfortable for the patients and because of that, it can also result in people accepting or declining. The other thing is: as we are combining the tests, I already know that for some of these, the performance is already quite good compared to the others. So there is a possibility that; I think that issue also came up with regards to the yield in regard to the sensitivity and specificity. With pooling sometimes you end up lowering the sensitivity and specificity instead of enhancing it. So that could also be a challenge, especially to the patients because as you are lowering the sensitivity and specificity then what it entails is that: for those who might be positive, some might have, some might end up being negative and then miss on the treatment as well as appropriate care they would have received. This one is a non-starter because microscopy has got the lowest. At the moment it has got the lowest sensitivity and specificity. If you try to compare with all the available TB testing techniques that are there, so that one is a non-starter; I think even with pooling, if they could know the sensitivity and specificity, I don't think it can go below 50%, whereby the microscopy I think would be around 25, it is quite low. Need the evidence that shows an increase in the yield and number of cases and also the evidence that shows that if the screening using pooled spacemen samples is far better compared to the other screening modalities, then that evidence should be able to compel us to adopt it. I don't think I can have any thoughts now because we were given the opportunity to go through all the protocol and make recommendations. But what I would say is: I realized that there are two components to this. There is the diagnostic aspect and then there is also the cost benefit analysis of these combinations. So, from the program's perspective or program point of view, we will be looking forward to seek this because it will work out in terms of the pooling and if it works out, what are the cost implications? if you can be able to show us that the cost is even lower compared to the conversional methods that we are using, then it is also another thing that can drive us into adopting the result from the study and make sure that the influencing policy of the program

- So I was just telling you about asymptomatic positives, so it's so easy to mix those kinds of patients, if it's really a human error. So I think that's just the negative side that I look at. If you are not lucky to pick the right specimen or pick the right whatever from the pool, you can easily say they are all negative and that will be false negative. Yes, we have discussed so many things but for me, the most important combination testing is the way to go, its doable and workable. It will be cost effective, efficient and if done very well it may also be a relief especially when i look

at the pool, it may be a relief to our health care providers. It may also reduce the breakdown of some of our equipment so I feel it’s a way to go and through community engagement, we can sustain it.

- Yeah, this particular pool testing has not been there, but I think if it is adopted, I think it gives also gifting, because this measures also the sensitivity of the test all together. When you pool every other then you test it, all the molecular diagnostics will be subjected to that particular testing. And once it is positive, you now subject it to the other so it gives issues of sensitivity and specificity. So this particular issue is of paramount. The only problem with it is issues of a lot of issues, because you have to now subjecting, at least subjecting to this particular test, subjecting to this particular test, it takes some slight time, but not much actually, since we know that the gene expert also takes maximum of 120 minutes for it to be tested, that's about two hours maximum. And also depends on how we do this. But I think the pool testing is paramount, more especially in decision making, and also when you go for outreaches and these things are done, definitely it also helps in trimming down to see the most sensitive molecular diagnostic at that particular area that you carry an outreach. Just like we said, individual different, genetic methods differs and etc. Yeah, these particular pool test can be done during the passive, can also be done during the active case finding, because what you require is for all the healthcare providers that will be involved in the active case finding to understand the step by step use of the issues of the brain testing, and I think it can be applied as well, and at the same time, it also gives an information, just like we said earlier, with issues of trying to find out exactly who actually have the disease and which particular test is also of value at this, but at the end, when there are divergent results coming from different molecular diagnostic tests. Then again, it requires the clinicians to give a time breaker regarding to that and take a decision.

- So pool testing is cost effective. Pool testing is cost effective in the sense that if you do the cost effectiveness, when you pool, maybe in 10s, in 20s, and you do and all of them becomes what negative you have handled that area. So those ones you won't need to do them one after the other. And in a situation where a particular batch becomes positive that you need to now retest them individually. One is based on probability, It is possible the first test you do, maybe the one that is positive, maybe the last one. But you still need to do them, do for all of them too, so, but if you look at it in cost effectiveness, even I think that is the way to go in terms of presumptive. In fact, the laboratory need to start looking at that because the cost per test is high and for sustainability, especially when this donor funded whatever will be over, we need to have, like our own platform, a way of doing things, because if we do a pool testing, it will help us a lot to cut costs. That's the truth. So pool testing for me, I will advise on this you can do a pool testing, the outcome will be a better cost effective.

- It's cost effective, know that, and it works better for us, because there's I don't want to believe that, but we are resource constrained country, so the little money we have pooling has been shown to work and if you have limited resources, rather than testing everybody, you can pool your samples, and with the historic experience that TB is not, the prevalence of TB in a particular place is not as high as that for malaria, it not as high as so it's, it works in our environment. It has been tested and proven to work. So it is a good one. It's a good method. In other countries, they have deployed it, and it has worked for them, even Nigeria. We have studies that have been done in Nigeria that is very cost effective, and it gives good results.

- I believe the main stakeholders are, first, the National Tuberculosis Program, and second, partners, especially the suppliers of these tests combination, such as CRP tests or pooled sample tests to reduce costs for Xpert testing. To implement this, the testing system, for example, in the tests combination, also includes screening tests. One of the current advantages is that both the government and the global community are strongly promoting AI, and the application of AI in X-ray diagnosis will also be significant. I hope that in the coming period, there will be breakthroughs and regulations that allow us to apply this in practical implementation. Currently, it is still being applied as a project, and there are no official regulations from the government that permit the use of AI for human testing to a certain extent; this is still lacking, and this applies not only in Vietnam but in many countries.
