# Supplementary material for: Feasibility and acceptability of pooled sputum testing for tuberculosis: a multicountry qualitative study of the Start4All project
Source: BMJ Open. 2026 Jul 30;16(7):e120434. doi: 10.1136/bmjopen-2026-120434 (PMC13436116; doi:10.1136/bmjopen-2026-120434)
Supplement: Supplementary file 2 [file bmjopen-16-7-s002.pdf]

Supplementary Table 1. Standards for Reporting Qualitative Research (SRQR) Checklist

O'Brien B.C., Harris, I.B., Beckman, T.J., Reed, D.A., & Cook, D.A. (2014). Standards for reporting qualitative research: a synthesis of recommendations. *Academic Medicine, 89(9)*, 1245-1251.

No.	Topic	Item (how this study addresses the SRQR standard)
**Title and abstract**		
S1	Title	Clearly identifies the study as a qualitative exploration of pooled testing for TB diagnosis across seven high-burden countries.
S2	Abstract	Provides a structured summary including background, objectives, qualitative methods (KIIs and FGDs), frameworks used, key findings, and conclusions.
**Introduction**		
S3	Problem formulation	Describes global TB diagnostic challenges and the evidence gap on the feasibility and acceptability of pooled testing; situates the study within WHO and Unitaid priorities.
S4	Purpose or research question	States the aim to explore stakeholder perspectives on feasibility and acceptability of pooled testing to inform future policy and guideline development.
**Methods**		
S5	Qualitative approach and research paradigm	Uses a descriptive qualitative design grounded in implementation science; analysis guided by Klaic, Barker, and French frameworks for feasibility, and Sekhon's framework for acceptability.
S6	Researcher characteristics and reflexivity	Research team includes TB, public health, and qualitative experts. Reflexivity maintained through debriefings and memos to address potential bias linked to researchers' involvement in Start4All.
S7	Context	Conducted within the Unitaid-funded Start4All initiative across seven WHO high-burden countries (Bangladesh, Brazil, Cameroon, Kenya, Malawi, Nigeria, Viet Nam) to capture diverse programme contexts.
S8	Sampling strategy	Purposive sampling of stakeholders (programme staff, healthcare providers, people with TB, and international experts) guided by data saturation; country teams ensured diversity.
S9	Ethical issues pertaining to human subjects	Ethical approval obtained from WHO ERC, LSTM REC, and national committees; written informed consent secured from all participants with guardian consent for minors.

S10 Data collection methods	KIIs and FGDs conducted from October 2024 to June 2025 by trained researchers to capture both expert and community perspectives; methods selected for depth and triangulation.
S11 Data collection instruments and technologies	Semi-structured topic guides based on feasibility and acceptability frameworks; piloted, refined through debriefings, translated for contextual and cultural clarity, and audio-recorded.
S12 Units of study	Included 6 international KIIs, 32 national KIIs, and 18 FGDs (with healthcare providers and people with TB) across seven countries.
S13 Data processing	Recordings transcribed verbatim, translated into English where needed, anonymised, verified for accuracy, and imported into NVivo 14 for analysis.
S14 Data analysis	Framework analysis combining deductive (predefined domains) and inductive (emerging insights) coding; cross-country comparison to identify shared and divergent themes.
S15 Techniques to enhance trustworthiness	Applied triangulation across data sources, reflexive practice, peer debriefings, audit trail documentation, and iterative refinement of topic guides and frameworks.
**Results/Findings**	
S16 Synthesis and interpretation	Findings structured by feasibility and acceptability domains, integrating cross-country insights on barriers, enablers, and contextual factors influencing pooled testing.
S17 Links to empirical data	Empirical evidence presented through direct participant quotes (KIIs and FGDs) illustrating core themes and contrasting views.
**Discussion**	
S18 Integration with prior work, implications, transferability, and contribution(s) to the field	Discusses alignment with prior pooled testing and TB diagnostic studies; identifies new insights on implementation, scalability, and acceptability across diverse health systems
S19 Limitations	Acknowledges variability in pooled-testing experience across countries, reliance on self-reported data, and limited observation; outlines mitigation through triangulation and reflexivity.
**Other**	
S20 Conflicts of interest	Authors declare no competing interests.
S21 Funding	Study funded by Unitaid (Grant 2022-50-START-4-ALL); funder had no role in design, data collection, analysis, or manuscript preparation.

Supplementary Table 2. Institutional Review Board approval numbers

Area	Ethical Board	REC Reference	Approval date
Overall	LSTM REC	22-084	14/11/2023
	WHO ERC (master protocol)	ERC.0003921	09/06/2023
Bangladesh	WHO ERC	ERC.0004062	10/06/2024
	icddr,b RRC (Research Review Committee)	PR-23002	22/01/2023
	LSTM REC	N/A	09/07/2024
Brazil	WHO ERC	ERC.0004061	27/06/2024
	Comissão Nacional De Ética Em Pesquisa (National Health Research Ethics Committee (CONEP))	68398523.9.0000.5546 / 6.138.117	23/06/2023
	Secretaria Municipal de Saúde	5800.22221.2023	28/02/2023
	LSTM REC	N/A	18/07/2024
Cameroon	WHO ERC	ERC.0004014	13/11/2023
	NEC (National Ethics Committee)	2023/04/1537	03/05/2023
	LSTM REC	N/A	14/11/2023
Kenya	WHO ERC	ERC.0004079	24/06/2024
	KEMRI (Kenya Medical Research Institute)	KEMRI/RD/22	17/07/2023
	LSTM REC	N/A	29/03/2023
Malawi	WHO ERC	ERC.0004033	16/01/2024
	COMREC (College of Medicine research and ethics committee)	P.01/23/3940	03/04/2023

	LSTM REC	N/A	12/08/2024
Nigeria	WHO ERC	ERC.0004072	04/06/2024
	NHREC (National Health Research Ethics Committee)	NHREC/01/01/2007	21/12/2022
	LSTM REC	N/A	10/01/2024
Vietnam	WHO ERC	ERC.0004084	24/06/2024
	National Lung Hospital ECBR (Ethics Committee for Biomedical Research)	66-23/ CN-HDDD-BVPTU	24/11/2023
	LSTM REC	N/A	28/06/2024

Supplementary Table 3. Demographics of the healthcare providers taking part in the FGDs

	Bangladesh	Brazil	Cameroon	Kenya	Malawi	Nigeria	Vietnam
**N**	7	6	8	6	10	8	25
**Gender (F/M)**	1/6	4/2	2/6	4/2	9/1	6/2	14/11
**Age (mean, ± SD, (range)**	34.7 ± 3.9 (31–42)	43.5 ± 5.8 (38–53)	43.8 ± 12.5 (25–65)	37.2 ± 12.9 (28–59)	44.6 ± 6.8 (27–50)	43.8 ± 10.0 (30–58)	41.4 ± 7.6 (25–60)
**Education* (N (type))**	6 (Q),1 (T)	6 (T)	2 (P), 2 (S), 2 (T), 1 (Q), 1 (U)	6 (T)	6 (T), 4 (S)	6 (T), 2 (S)	25 (T)
**Occupation** (N (type)) – NTP staff**	2 (M), 1 (T), 1 (O)	1 (T), 1 (D)	2 (T), 2 (O)	3 (N), 1 (D), 1 (T), 1 (R)	3 (D)	1 (D), 2 (O)	6 (T)
**Occupation** (N (type)) – Non-NTP staff**	3 (O)	4 (N)	3 (N), 1 (O)	-	1 (T), 1 (M), 1 (L), 4 (O)	1 (M), 4 (L)	9 (N), 8 (D), 2 (T)

*** Education; U= unknown, P= primary, S= secondary, T= tertiary, Q= quaternary**

**** Occupation, N=nurse, D=doctor, T= technician, R=radiographer, M= medical officer, L= lab officer, O=other health care related occupation**

Supplementary Table 4. Demographics of the people with TB taking part in the FGDs

	Bangladesh	Brazil	Cameroon	Kenya	Malawi	Nigeria	Vietnam
**N**	7	7	5	8	8	7	6
**Gender (F/M)**	4/3	2/5	1/4	3/5	1/7	3/4	1/5
**Age (mean, ± SD, (range)**	47.4 ± 15.6 (33–78)	44.3 ± 15.9 (24–70)	35.0 ± 7.1 (25–45)	31.3 ± 13.5 (20–60)	40.0 ± 9.8 (24–54)	38.7 ± 13.6 (15–58)	48.2 ± 15.5 (25–69)
**Education* (N (type))**	3 (Q), 2 (N), 2(P)	6 (S), 1 (N)	3 (P), 1 (S), 1 (U)	4 (T), 3 (S), 1 (N)	3 (P), 5 (S)	6 (S), 1 (P)	6 (U)
**Occupation ** (N (type))**	4 (H), 1 (T), 2 (D)	2 (H), 1 (C), 1(U), 2 (M), 1 (O)	3 (F), 1 (T), 1 (C)	3 (E), 2 (S), 1 (U), 2 (O)	2 (U), 4 (D), 1 (F), 1 (O)	3 (O), 2 (R), 1 (B), 1 (S)	3 (E), 1 (U), 2 (K)

*** Education; N=none, U= unknown, P= primary, S= secondary, T= tertiary, Q= quaternary**

****Occupation; H=housewife, T=teacher, F=farmer, E=self-employed, S=student, U= unemployed, D= day laborer, K= unknown, C=carpenter, M=mason, B=builder, R=trader, O=other**

Supplementary Table 5. Demographics of the Key Informant Interview (KIIs) participants

	Bangladesh	Brazil	Cameroon	Kenya	Malawi	Nigeria	Vietnam	International
**N**	4	4	6	4	4	5	5	6
**Gender (F/M)**	0/4	3/1	3/3	2/2	1/3	2/3	2/3	4/2
**Age (mean, ± SD, (range)**	43.0 ± 6.1 (34–51)	42.0 ± 11.0 (30–60)	41.2 ± 7.5 (25–54)	33.8 ± 7.3 (24–40)	39.3 ± 4.7 (35–45)	51.0 ± 7.0 (38–58)	46.4 ± 12.0 (27–58)	–
**Education* (N (type))**	4 (T)	4 (T)	6 (T)	4 (T)	4 (T)	5 (T)	5 (T)	6 (U)
**Occupation (N (type))**	Programme and Policy Leadership	Programme and Policy Leadership	5 Programme and Policy Leadership; 1 Technical Specialist	3 Clinical and Public Health Professionals; 1 Laboratory Specialist	Programme and Policy Leadership	4 Programme and Policy Leadership; 1 Laboratory Specialist	2 Programme and Policy Leadership; 1 Clinical Professional; 1 Laboratory Specialist; 1 Community Representative	Technical and Implementation Specialists

*** Education; U= unknown, P= primary, S= secondary, T= tertiary, Q= quaternary**
