# Supplementary material for: Feasibility and acceptability of pooled sputum testing for tuberculosis: a multicountry qualitative study of the Start4All project
Source: BMJ Open. 2026 Jul 30;16(7):e120434. doi: 10.1136/bmjopen-2026-120434 (PMC13436116; doi:10.1136/bmjopen-2026-120434)
Supplement: Supplementary file 3 [file bmjopen-2026-120434.reviewer_comments.pdf]

PEER REVIEW HISTORY

BMJ Open publishes all reviews undertaken for accepted manuscripts. Reviewers are asked to complete a checklist review form and are provided with free text boxes to elaborate on their assessment. These free text comments are reproduced below.

ARTICLE DETAILS

Title (Provisional)

Feasibility and acceptability of pooled sputum testing for tuberculosis: a multi-country qualitative study of the Start4All project

Authors

Iem, Vibol; Vo, Luan Nguyen Quang; Codlin, Andrew; Iwakun, Mosunmola; Kwanjo Banda, Chimwemwe; Okelloh, Douglas Akunda; Mbuli, Cyrille Ndamnsah; Dosea, Aline; Santos , Victor Santana; Chowdhury, Kamal Ibne Amin; Garg, Tushar; Creswell, Jacob; Ringwald, Beate; Cubas Atienzar, Ana I; Byrne, Rachel L; Wingfield, Tom; On behalf of, The Start4All investigators

---

VERSION 1 - REVIEW

Reviewer **1**

Name **Xin, Henan**

Affiliation **Chinese Academy of Medical Sciences & Peking Union Medical College Institute of Pathogen Biology**

Date **02-May-2026**

COI **None**

The Author explored the feasibility and acceptability of pooled sputum testing for tuberculosis using a multi-country qualitative study design. It provides insightful qualitative evidence to support the scale-up and policy formulation of pooled sputum testing.The description of data processing and coding is generally clear, but several key methodological details need to be supplemented to improve methodological rigor. It is recommended to:

1.Clearly state the specific qualitative analytical approach (e.g., thematic framework analysis).

2.Add details about double independent coding, inter-coder reliability test, and consensus resolution of coding discrepancies.

3.Supplement quality control measures for translation, such as back-translation.

4.Briefly describe the principle of data saturation and researcher reflexivity to enhance trustworthiness of the qualitative findings.

**Reviewer**	**2**
**Name**	**Church, E**
**Affiliation**	**Oregon Health & Science University, Division of Infectious Diseases**
**Date**	**25-Jun-2026**
**COI**	**None**

Overall a good quality study outlining needed information about implementation of a new testing structure. The inclusion of many diverse stakeholders makes this a strong paper.

Intro:

Line 7 page 6 citation 1: Global TB report 2025 available, recommend updating numbers

Line 36 page 6: “report over 90% agreement”, can this be given as a specific range, and broken down into sensitivity and specificity? These specific numbers are quite important for acceptability of the method

Methods:

No comments

Results:

Line 9-10 page 12: Did participants from the lab identify a threshold for “generally low” TB positivity rates? That statement may be very setting specific, as low by a north American or western European standard may be very different than low in the context of a high incidence country

Line 16-17 page 13: Indicates some concern about lowering sensitivity. Have any countries implemented a system where individual testing can be requested instead of pooling based on clinician concern?

Discussion:

Line 13 page 16:

VERSION 1 - AUTHOR RESPONSE

I. Reviewer 1

1. Clearly state the specific qualitative analytical approach (e.g., thematic framework analysis).
   Response: We have revised the Methods section (Qualitative approach and

conceptual framework research paradigm), to explicitly state that the study used a thematic framework analysis. The text has been updated at line 57-59 as follows: *"Data were analysed using a thematic framework analysis. The analysis was guided by an integrated conceptual framework, which combined established feasibility and acceptability frameworks adapted to the scenario of pooled testing."*

2. Add details about double independent coding, inter-coder reliability test, and consensus resolution of coding discrepancies.
   Response: We have revised the Methods section (Data processing and analysis) to clarify the coding process. The text has been updated at lines 157-163 as follows: *"All transcripts were imported into NVivo 14 for coding. Country research teams initially coded transcripts using a predefined codebook aligned with the feasibility and acceptability frameworks. The first author (VI) then reviewed and recoded transcripts where necessary to ensure consistency across countries. Regular consensus meetings with the country qualitative research focal points were held throughout the analysis to discuss coding decisions, resolve discrepancies, and iteratively refine the codebook, thereby promoting consistent application of the coding framework across countries."*
   We did not undertake formal inter-coder reliability testing, as our thematic framework analysis emphasised interpretive consensus rather than statistical agreement.

3. Supplement quality control measures for translation, such as back-translation.
   Response: Back-translation was not undertaken as part of the study. The manuscript describes the translation procedures at line 152-156 as follows*: "All audio recordings were transcribed verbatim in the original language by trained research staff. In Bangladesh, Brazil, Cameroon, and Viet Nam, transcripts were professionally translated into English, preserving the original meaning. Each transcript was reviewed for accuracy and anonymised, removing any identifying information to ensure participant confidentiality."*

4. Briefly describe the principle of data saturation and researcher reflexivity to enhance trustworthiness of the qualitative findings.
   Response: We have revised the Methods section and added the following text to describe:
   - data saturation at lines 112-114: *"Sampling continued until thematic saturation was reached, assessed through ongoing review of interview and discussion data*

to determine the point at which no new themes relevant to feasibility and acceptability emerged from additional FGDs or KIIs."
- researcher reflexivity to enhance trustworthiness at line 71-82: *"The research team included individuals with expertise in TB diagnostics, implementation science, and qualitative research. Several members, including the corresponding author and senior author, had prior experience with pooled testing and were generally supportive of its potential benefits, which provided contextual insight but also posed a risk of positive bias. To mitigate this, data collection tools explicitly probed both perceived advantages and concerns, interviewers actively sought dissenting views, and coding was undertaken by analysts with varying levels of involvement in pooled testing. Regular multidisciplinary discussions throughout data analysis encouraged researchers to critically reflect on how their prior experience and assumptions might influence interpretation, and to assess whether emerging interpretations were supported by the data, thereby enhancing the trustworthiness of the qualitative findings. These measures aimed to ensure that analysis was grounded in participants' perspectives rather than researchers' prior expectations."*

II. Reviewer 2

1. Line 7 page 6 citation 1: Global TB report 2025 available, recommend updating Numbers
   Response: The manuscript already cites the latest available WHO Global Tuberculosis Report (2025). As the WHO Global Tuberculosis Report 2025 presents TB burden estimates for 2024, the figures reported in the manuscript (10.7 million people developing TB and 1.23 million TB deaths in 2024) are the most recent available. Estimates for 2025 are not yet available and will be reported in the WHO Global Tuberculosis Report 2026. Therefore, no changes to the manuscript were required.

2. Line 36 page 6: "report over 90% agreement", can this be given as a specific range, and broken down into sensitivity and specificity? These specific numbers are quite important for acceptability of the method
   Response: We have revised the sentence to report the diagnostic performance more precisely by replacing the statement "over 90% agreement" with the reported ranges of sensitivity and specificity from the published evidence. The text has been revised at line 22-23 as follows: *"Studies evaluating pooled sputum testing using molecular diagnostics have reported sensitivities of 87.5%–98%, depending on pool size and assay used, and specificities of 99%–100%*

compared with individual testing, alongside improved efficiency and substantial cost savings."

3. Line 9-10 page 12: Did participants from the lab identify a threshold for "generally low" TB positivity rates? That statement may be very setting specific, as low by a north American or western European standard may be very different than low in the context of a high incidence country
   Response: Participants did not identify a specific TB positivity threshold at which pooled testing would no longer be appropriate. The text has been revised at line 209-211 as follows: *"Laboratory managers and staff emphasised that pooled testing works best in facilities where TB positivity rates are generally low, allowing laboratories to maintain both accuracy and efficiency, although no specific positivity threshold was identified."*
   The Discussion already highlights that the value of pooled testing is context dependent and should be guided by local epidemiological and operational conditions at line 319-324: *"Stakeholders viewed pooled testing as strategically valuable only under specific epidemiological and operational conditions. Its efficiency gains were perceived to depend on low TB positivity rates and manageable testing volumes, aligning with modelling evidence showing diminishing returns above approximately 30% positivity. This indicates that pooled testing is not inherently superior to individual testing and its value is context dependent".*

4. Line 16-17 page 13: Indicates some concern about lowering sensitivity. Have any countries implemented a system where individual testing can be requested instead of pooling based on clinician concern?
   Response: We have addressed this point in the Discussion by noting that selective implementation strategies can help mitigate concerns about reduced diagnostic sensitivity. The following statement has been added at line 340-345: "*In practice, concerns about reduced sensitivity can be addressed through selective implementation strategies. For example, the Cameroon National TB Programme pooled testing strategy explicitly excludes people living with HIV and people previously treated for TB from pooled testing, with these individuals instead undergoing individual molecular testing as their initial TB test."*
